# Microenvironments on individual sand grains enhance nitrogen loss in coastal sediments

**DOI:** 10.1038/s41598-025-00755-3

**Published:** 2025-05-11

**Authors:** Farooq Moin Jalaluddin, Soeren Ahmerkamp, Hannah K. Marchant, Volker Meyer, Klaus Koren, Marcel M. M. Kuypers

**Affiliations:** 1 Max Planck Institute for Marine Microbiology, 28359 Bremen, Germany; 2 Leibniz Institute for Baltic Sea Research Warnemünde, 18119 Rostock, Germany; 3 MARUM-Center for Marine Environmental Sciences, University of Bremen, 28359 Bremen, Germany; 4 Department of Biology, Aarhus University Centre for Water Technology, Aarhus University, Aarhus, 8000 Denmark

**Keywords:** Permeable sediments, Aerobic denitrification, Microniches, Oxic-anoxic interface, Nitrogen cycling, Carbon cycle, Element cycles, Ocean sciences

## Abstract

The permeable silicate sediments which cover more than 50% of the continental shelves are a major, but poorly constrained sink for the vast amount of anthropogenic nitrogen (N) that enters the ocean. Surface-attached microbial communities on sand grains remove fixed-N via denitrification, a process generally restricted to anoxic or low oxygen (O_2_) environments. Yet, in sands, denitrification also occurs in the centimeters thick well-oxygenated surface layer, which leads to additional and substantial N-loss. So far however, the underlying mechanisms that drive denitrification in oxic sands are poorly resolved. In this study, we applied a non-invasive microfluidic technique to visualize and quantify how sediment-attached microorganisms shape O_2_ availability on the surface of silicate sand grains. This revealed a remarkable heterogeneity in rates; with colonies of O_2_ consuming and producing microorganisms situated within micrometers of each other. Using a mechanistic approach to model respiration on the surface of a single silicate sand grain we showed that the high rates of O_2_ consumption within the microbial colonies on the sand-grain surface outpace O_2_ supply from the surrounding pore water. As a result anoxic microenvironments develop on the sand grain surface, which so far have been invisible to conventional techniques. The model results indicate that anaerobic denitrification occurring in these anoxic microenvironments can account for up to 74% of denitrification in oxygenated sands, with the remainder occurring in the presence of oxygen. In a preliminary upscaling approach, using a global dataset we estimated that anoxic microenvironments in oxygenated surface layers could be responsible for up to a third of the total N-loss that occurs in silicate shelf sands. Consequently, denitrification in anoxic microenvironments drives substantial anthropogenic-N removal from continental silicate shelf sands.

**Supplementary Information:**

The online version contains supplementary material available at 10.1038/s41598-025-00755-3.

## Introduction

Permeable sands cover more than half of the continental shelf seafloor where they function as biocatalytic filters^1,2^, removing vast amounts of the anthropogenically derived nitrogen (N) that reaches coastal seas via riverine and groundwater discharge^3–7^. The extensive N-loss that occurs in sands is driven by highly active sediment-attached microorganisms which are constantly re-supplied with substrates (i.e. nitrate and organic matter) due to the advective flow of seawater through the pore space between individual sand grains^5,6,8^. These microbial communities remove fixed-N from the environment via denitrification, a process which is typically restricted to anoxic or low oxygen (O_2_) environments^9,10^. Yet, in sands, denitrification has been measured when O_2_ concentrations within the sediment are high^7,8,11,12^. This so-called aerobic denitrification has the potential to greatly enhance the volume of sediment in which N-loss can occur, as O_2_ can penetrate centimeters deep into sandy sediments dependent on pore water flow velocity^13–16^. Yet, despite the potential importance of aerobic denitrification, so far, the underlying mechanisms that drive it are still not understood.

Almost all known denitrifying microorganisms are facultative anaerobes, i.e. they can also respire O_2_. However, cultured denitrifiers typically downregulate the transcription and synthesis of denitrification enzymes in the presence of O_2_, as from a bioenergetic and kinetic perspective, O_2_ is a more favorable electron acceptor^9^. However, in sandy sediments, microorganisms seem to constantly transcribe both aerobic and denitrification respiration pathways as a response to the rapidly changing availability of O_2_^11^. Thus, previously, aerobic denitrification in sandy sediments has been attributed to denitrifying communities performing aerobic and anaerobic respiration at the same time^8,11,17^. Additionally, the presence of anaerobic microbial activity in seemingly oxic environments can be a result of the formation of anoxic microenvironments due to biofilm formation^18,19^. When bacteria colonize surfaces in thick layers (i.e. biofilms), their respiratory activity can lead to the establishment of O_2_ gradients, allowing anaerobic processes to occur even when they are surrounded by well-oxygenated waters^20–22^. This can be exacerbated in soils and sandy sediments, where the pore spaces through which water flows can be clogged by biological processes such as extensive biofilm growth^23–26^, trapping of aggregates^20,27^, streamers^28,29^, and tortuosity effects^30,31^. However, microbial colonization on marine sand grains seems to be typically limited to monolayers of microorganisms, which are restricted to cracks and depressions on the sand grain surface^32–34^. Despite the microscale size of these sand grain surface microenvironments, diffusion limitation has still been hypothesized to occur within them^35^, potentially leading to the formation of anoxic microenvironments, but so far this has never been observed. This might be a direct consequence of the fact that the techniques frequently applied to study O_2_ dynamics in biofilms are unable to resolve O_2_ gradients which occur within the diffusive boundary layer (DBL) surrounding single sand grain surfaces. Thus, we have been unable to gain a mechanistic understanding of how sand grain attached microorganisms shape the microenvironment around them and control the potential for anaerobic processes to occur.

Here, we visualized for the first time the microbial distribution and volumetric O_2_ consumption/production rates at the surface of single silicate sand grains, using phosphorescent O_2_ sensitive nanoparticles. To assess how microscale heterogeneity of O_2_ consumption and production at the sand grain surface propagates into the DBL around the sand grain and the surrounding bulk pore water, we developed a multiphysics model for a silicate sand grain. Subsequently, we investigated how these micro-scale processes affect N-loss from continental silicate shelf sediments.

## Results and discussion

### Patchy colonization of sand grain surfaces by photo- and non-phototrophic microorganisms

**Fig. 1:**
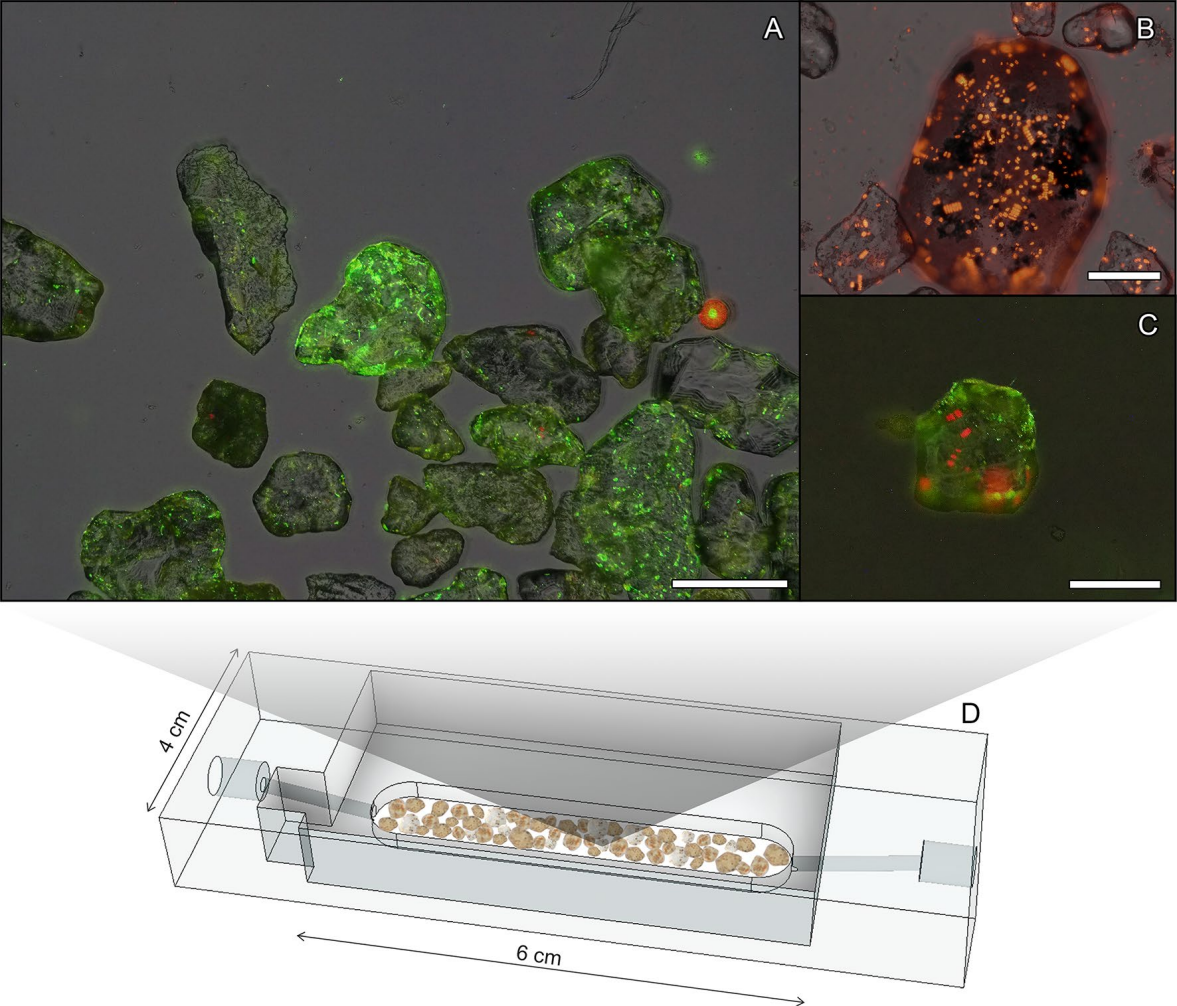
Micrographs showing the colonization of single sand grains by microorganisms. (**A**) dsDNA staining (SYBR Green I, green, exc.:469/35 nm, em.: 510/42 nm) overlaid with RGB image of sand grains revealing the heterogeneous microbial colonization on individual sand grains, (**B**) autofluorescence image (red) showing colonization of chlorophyll-*a* containing phototrophic microorganisms (exc.: 469/35 nm, em.: > 590 nm), (**C**) overlay of dsDNA staining (green) and autofluorescence image (red) reveals co-colonization of photo- and non-phototrophic microorganisms on the sand grains. (**D**) Conceptual view of the acrylic microfluidic device filled with sand grains to conduct the incubations. See also Table S1. Scalebars denote 250 μm.

We used fluorescence microscopy and SYBR Green I staining to visualize and quantify the distribution of microorganisms on the surface of single sand grains (Fig. 1A), which were collected from an intertidal region in the North Sea characterized by high microbial O_2_ respiration and N-loss rates^5,6,36,37^. Microscopic imaging of cells revealed a sand surface colonization of around 1.3⋅10^8^ cells cm^−3^ (± 0.4⋅10^8^ cells cm^−3^) of sediment, which occurred in patches of thin monolayers (Fig. 1A, Table. S1). Although our visualization revealed that microorganisms tended to colonize the sand grain surface in patches, they also showed that microorganisms did not form the 3D-structured thick biofilms that are known to cause diffusion limitation^18–20^, pore-space clogging^24^ and the formation of large anoxic microniches.

To distinguish between potential photosynthetic and non-photosynthetic O_2_-respiring microorganisms on the sand surface we imaged the autofluorescence (exc.: 469/35 nm, em.: > 590 nm) from chlorophyll-*a* containing microorganisms, i.e. microphytobenthos^38,39^ (Fig. 1B). Correlative imaging with SYBR Green I staining showed that individual sand grains were diversely co-colonized by primary producers and other non-chlorophyll containing, likely O_2_ respiring organisms (Fig. 1C). Overall, cell numbers of photosynthetic microorganisms were substantially lower than those of non-photosynthetic microorganisms (3.5⋅10^6^ cells cm^−3^ of sediment compared to a total of 1.3⋅10^8^ cells cm^−3^ of sediment). These cell counts of non-photosynthetic organisms compare well to those determined previously in sandy sediments^40–43^.

The morphology of the autofluorescent photosynthetic microorganisms indicated that they were likely cyanobacteria with typical sizes of < 1 μm as well as coccoid and disc-shaped diatoms which were approx. 80 μm large. These results fit to metagenomic analyses of the microbial community composition of photosynthetic microorganisms associated with intertidal sediments from the North Sea (Fig. S3). Based on the classification of small subunit (SSU) ribosomal RNA genes within the sand flat metagenome, Cyanobacteria (predominantly from the orders *Cyanobacteriales* and *Synechococcales*) comprised ~ 2% of the total bacterial community. Diatoms, particularly from the classes *Bacillariophyceae* and *Mediophyceae* comprised more than 96% of the reads associated with photosynthetic eukaryotes (Fig. S3 B). The presence of these firmly attached phototrophic microorganisms indicates that the sand grains were regularly exposed to light in situ, likely due to sediment redistribution and bedform migration^13^, as light only penetrates the top few millimeters of silicate sands^44^. This dynamic environment not only supports phototrophs but also shapes a diverse microbial community in which denitrification is not confined to a single taxonomic group. Instead, the capacity for denitrification appears to be a widely shared functional trait selected for across the community^11^. Thus, we did not visualize individual taxa attached to the sand grains (as in^34^). However, the analysis of the abundance of denitrification genes relative to a housekeeping gene (*rpoB*) within the sediment, indicated that around 15–20% of the microbial community within the sediments has the potential to denitrify (Fig. S4). Taken together the microscopic imaging showed a highly heterogeneous colonization on the surface of individual sand surfaces.

### Co-occurring O_2_ consumption and production on individual sand grains

**Fig. 2:**
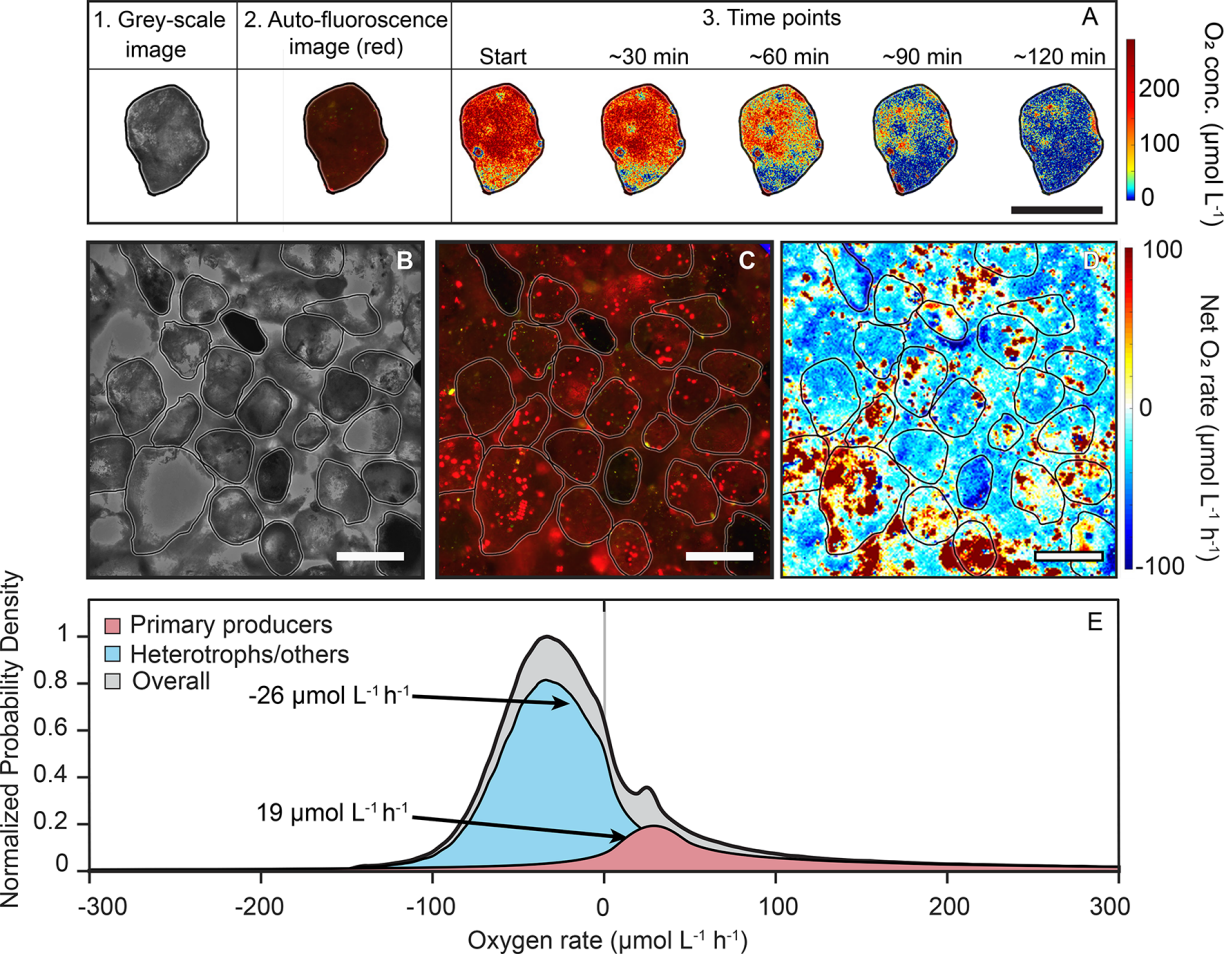
Microbial colonization of silicate sand grain surfaces and associated zones of O_2_ production and consumption that result in highly heterogeneous O_2_ concentrations at the surface of sand grains. (**A**) Development of heterogenous O_2_ concentrations on the surface of single sand grain over time. A.1 grey scale image of a sand grain. A.2 Auto-fluorescence (exc.: 469/35 nm, em.: > 590 nm) as an indicator for oxygen-producing photosynthetic microorganisms. A.3 Heterogeneities in O_2_ concentrations developing over time at the sand grain surface. (**B**) Grey-scale image of silicate sand grains incubated within the microfluidic chip, (**C**) auto-fluorescence image (exc.: 469/35 nm, em.: > 590 nm) showing the location of photosynthetic microorganisms on the sand grain surfaces, (**D**) O_2_ consumption and production on the sand grain surface in the microfluidic chip (blue color indicates O_2_ consumption rate and red color indicates O_2_ production rate. (**B**–**D**) The same field of view. (**E**) Normalized density distribution of the O_2_ rate for microenvironments, depicted in grey. The O_2_ rates were subsequently classified based on whether photosynthetic microorganisms were present in the pixels where the rate was measured, with net production highlighted in red, and other microenvironments showing net consumption, indicated in blue. Arrows indicate the median O_2_ rates of 19 µmol L^−1^ h^−1^ and -26 µmol L^−1^ h^−1^ for the photosynthetic microorganisms and other microorganisms, respectively. Note that the O_2_ consumption could have been associated with heterotrophic remineralization of organic matter as well as chemolithotrophic processes such as nitrification. Scalebars denote 200 μm.

The sand grains were collected from an intertidal flat in a region which is characterized by high net O_2_ respiration and N-loss rates (e.g^36,37^). In line with this, we observed a similar high net O_2_ consumption as previous studies (~ 26 µmol O_2_ L^−1^ h^−1^^45^). To determine the O_2_-respiration by individual surface-attached microbial colonies, we developed a new microfluidic-based imaging method (Fig. 1D). Briefly, we coated sand grains with phosphorescent O_2_-sensitive nanoparticles and subsequently quantified the change in O_2_ concentrations over time. We carried out the experiments under low light conditions in order to quantify the impact of both the photo- and non-phototrophic organisms on the sand grains. We observed heterogeneities in O_2_ concentrations developing at the surface of individual sand grains within 30 min, with patches on the sand grain surfaces turning anoxic within one hour; even though O_2_ concentrations were still > 100 µmol O_2_ L^−1^ on adjacent parts of the sand grain (Fig. 2A and Fig. S5). Until now, these anoxic microenvironments on the surface of silicate sand grains escaped detection because they are too small to resolve using more conventional instruments like microsensors^46,47^. Imaged changes in O_2_ concentration were used to determine volumetric O_2_ production and consumption rates on micrometer scales (1–10 μm) (see methods for details). Net median volumetric O_2_ rates were approx. -20 µmol O_2_ L^−1^ h^−1^ but varied strongly from -46 µmol O_2_ L^−1^ h^−1^ (25% percentile) to 12 µmol O_2_ L^−1^ h^−1^ (75% percentile) (Fig. 2). Overall, our direct imaging approach revealed a mosaic of O_2_ consuming and producing patches situated within micrometers of each other on the surface of a single sand grain (Fig. 2B-D).

The patches of O_2_ production directly correlated to areas colonized by auto-fluorescent phototrophic O_2_-producing microorganisms, while the O_2_-consuming areas were non-fluorescent, most likely representing colonies of non-photosynthetic O_2_ respiring microorganisms (Fig. 2E). Both these functional groups of microorganisms are known to colonize sand grains^48–51^, however their ability to noticeably affect O_2_ gradients over such small scales has not been reported before. The chlorophyll-*a* containing photosynthetic patches had median O_2_ production rates of 19 µmol O_2_ L^−1^ h^−1^ (Fig. 2E, red). Whereas the regions without chlorophyll-a had a net O_2_ consumption rate of 26 µmol O_2_ L^−1^ h^−1^ (Fig. 2E, blue). The decrease in O_2_ concentration for the entire incubation time period (4 h) was non-linear, most likely caused by diffusion limitations (see Fig. S6). However, the net consumption values represent a conservative estimate due to non-linear decrease and as we observed O_2_ consumption in some of the masked areas containing primary producers.

### Mechanism leading to anoxic microenvironments and denitrification on single sand grains

**Fig. 3:**
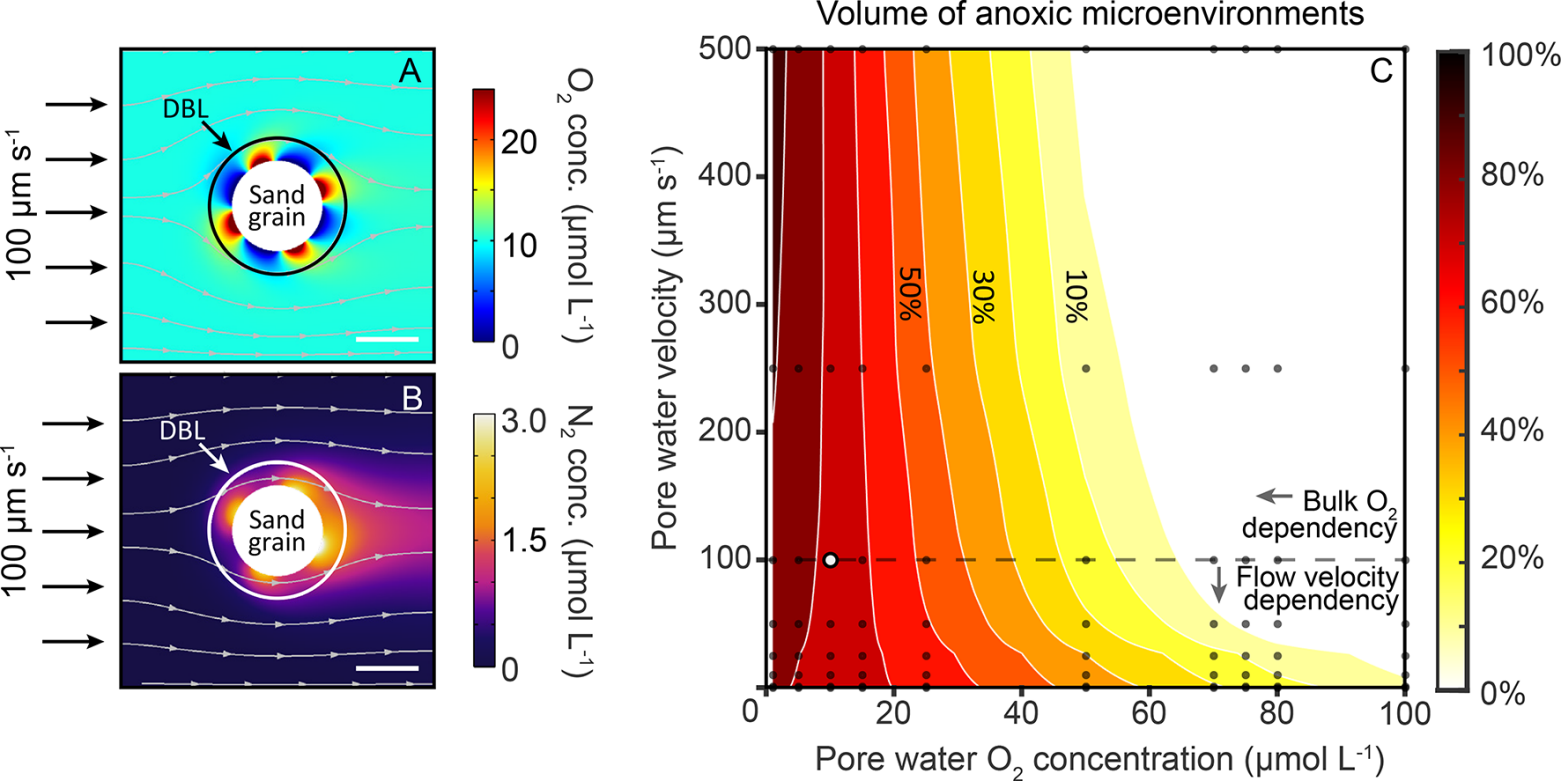
The development of anoxic microenvironments and associated N-loss on a modelled sand grain colonized with O_2_ consuming and producing microbial colonies. (**A**) Patchy distribution of oxygen (O_2_) producing and consuming microenvironments within the diffusive boundary layer (DBL) indicate the fast cycling of O_2_ on micrometer scales and the formation of anoxic microenvironments on the sand surface, despite the presence of bulk ambient O_2_. (**B**) Even though zones of production and consumption are in direct proximity, anoxic microenvironments are formed in the presence of O_2_ resulting in the production of nitrogen (N_2_) through denitrification. Streamlines indicate the flow field (scale bar in (**A**), (**B**) represents 200 μm). (**C**) Phase diagram indicating the anoxic volume (in which rates are taking place) in dependence to pore water velocities and inflow O_2_ concentrations (based on 80 model runs indicated through gray dots, white dot indicates model run depicted in (**A**), (**B**)). At lower flow velocities (below 10 μm s^−1^), the volume of anoxic microenvironments is strongly dependent on the flow velocities through which the boundary layer thickness (diffusive length scale) is determined. At higher flow velocities (above 100 μm s^−1^) the anoxic volumes mainly depend on inflow O_2_ concentrations. An additional 80 model runs were carried out where no O_2_ production was occurring to simulate a scenario with no photosynthetic activity, and showed similar results (Fig. S9 and supporting information text).

We derived a multiphysics model for a typical silicate sand grain (median diameter 290 μm, Fig. 3, Fig. S7) to assess how heterogeneity of O_2_ consumption and production at the sand grain surface propagates into the diffusive boundary layer (DBL) around the sand grain and the surrounding bulk pore water. First, we used the volumetric O_2_ consumption and production rates determined using the microfluidic-chip to model the O_2_ consumption/production rate heterogeneity at the sand grain surface (see methods). The O_2_ consumption and production rates on the microbially colonized surface of the sand grain were modeled to range between -955 mmol L^−1^ h^−1^ and 514 mmol L^−1^ h^−1^, corresponding to single cell O_2_-respiration and photosynthesis rates of 0.4 fmol O_2_ cell^−1^ h^−1^ and 7.1 fmol O_2_ cell^−1^ h^−1^, respectively (see methods). Subsequently, microbial colonies with net O_2_ production and consumption rates were distributed across the modelled sand grain surface (Fig. 3A), simulating the observed patchy distribution of microbial colonies, respiration and photosynthesis (Figs. 1 and 2). Finally, we varied pore water velocities and bulk O_2_ concentrations in the model to assess how O_2_ consumption/production rate heterogeneity at the sand grain surface propagates into the DBL and surrounding bulk pore water in the presence of pore water flow. A total of 1764 model runs, with pore water O_2_ concentrations from 1 to 100 µmol L^−1^ and pore water flow velocities of 0 to 500 μm s^−1^ were carried out to simulate the ranges observed in the environment^13,52–54^ (see Table S1 for summary of all parameters). The sand grain model results revealed that the variations in O_2_ concentrations resulting from O_2_ rate heterogeneity at the sand surface propagated into the DBL, but not into the surrounding pore water (Fig. 3A). Consequently, the DBL around individual sand grains had O_2_ concentrations which differed greatly from the surrounding pore waters, even when the pore waters were well ventilated. Furthermore, the modelling runs indicated that between 20 and 100% of the net O_2_-respiring non-photosynthetic microbial colonies on the sand grain surface turn anoxic when pore water O_2_ concentrations were below 50 µmol L^−1^ or when pore water velocities were lower than 100 μm s^−1^ (Fig. 3C). Under these conditions anoxic microenvironments formed as respiration rates outpaced the diffusion of O_2_ into the DBL (Fig. 3C). Intriguingly, we found that the activity of photosynthetic microbial colonies on the sand grain surface had only a minor effect on the volume of anoxic microenvironments, implying that the results are also applicable to non-illuminated silicate sandy sediments (see methods and supplementary information text). Sand grain surface roughness was not included in the main modeling results or sensitivity analysis, which makes our estimates on microenvironments conservative. To evaluate its potential effect, we conducted additional calculations in which surface roughness, represented as height deviations from a smooth surface was tested for its impact on DBL thickness at flow velocities between 10 and 100 μm s⁻¹ (see methods). This revealed that surface roughness, which was between ~ 2–35 μm could increase the DBL thickness by up to 20%. Such an increase would reduce diffusive exchange between the grain surface and surrounding pore water, thereby promoting the formation of anoxic microsites.

Subsequently, having identified when microbial colonies turn anoxic, we included a denitrification parametrization within the single sand grain model, using the ratio of denitrification rates to O_2_ consumption rates determined previously for sandy shelf sediments (Fig. S8, see methods). This robust empirical correlation between O_2_ consumption rates and denitrification rates under oxic and anoxic conditions, respectively, is derived from diverse sandy sediment studies^5,12,17,35,55,56^, and is a well-established proxy by which denitrification is determined in benthic modelling studies (e.g^57^. By applying this parameterization, we observed substantial N_2_ production at the surface of the sand grains with rates of 0.2 nmol N per day per anoxic microbial colony, summing to a total of 0.8 nmol N per day per sand grain, which diffused from the DBL into the porewater (Fig. 3B).

Overall, the model reproduced the observed microbial colonization and O_2_ dynamics of a single sand grain, and showed that, anoxic microenvironments form due to O_2_ consumption rates at the sand grain surface outpacing diffusive O_2_ supply from the surrounding oxic pore water (Fig. 3A). We confirmed that the development of anoxic microenvironments is a persistent feature in a total of 1764 model runs where O_2_ consumption rates in the microbial colonies, diffusion coefficients, flow velocity and microbial distributions were varied. It is likely that this mechanism of anoxic microsite formation is active throughout the oxic silicate sands of the continental shelves, as they are all exposed to similar physical forcing and flow conditions^2^ and furthermore, exhibit similar microbial communities^9,51,58–60^ and microbial colonization patterns^32,34,48^.

### The role of anoxic microenvironments in biogeochemical cycling in oxic sandy shelf sediments

**Fig. 4:**
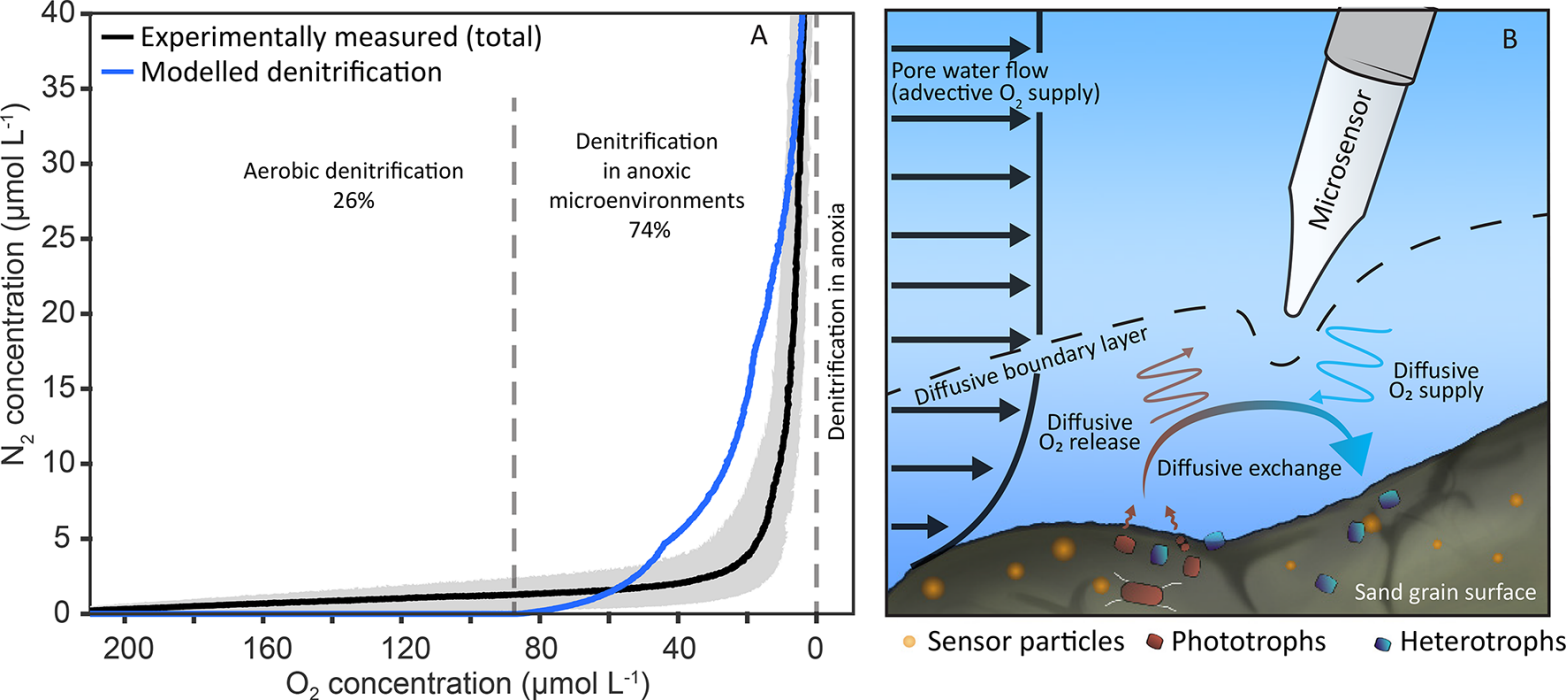
Modelled N_2_ production from anaerobic denitrification in anoxic microenvironments compared to experimental observations of N_2_ production in the presence of O_2_. (**A**) The relationships derived from the modeling outputs were applied to a previously published dataset^11^ where N_2_ production was measured simultaneously to oxygen respiration in sandy sediments (mean concentration as solid line and gray shaded area represents the concentration range from different experiments). This allowed an estimation of the proportion of sand grain surfaces which were anoxic for the given oxygen concentration; subsequently N_2_ production was calculated within these microenvironments based on the maximal anoxic rate of denitrification measured in the same incubation (blue solid line). Anoxic microenvironments started accumulating within the sediment even when bulk pore water still had an O_2_ concentration of 90 µmol L^−1^. The remaining N_2_ production could not be attributed to anoxic microenvironments and was likely produced in the presence of oxygen (data used can be found in Fig. S10). Based on the model relationships, aerobic denitrification contributes 26%, while denitrification in anoxic microenvironments accounts for 74% of denitrification under oxic conditions. (**B**) Reduction of porewater velocity around sand grains leads to a limited supply of substrate due to the formation of a diffusive boundary layer (DBL). When microbial respiration rates within the boundary layer exceed diffusion of O_2_ into the DBL, local spots of anoxia can form, even when O_2_ is produced nearby by O_2_ producing microorganisms. Conventional techniques such as micro-sensors would not be able to resolve these micrometer scale patches of anoxia as they disrupt the DBL which leads to mixing between the anoxic and oxic microenvironments as well as the surrounding waters.

The diffusive O_2_ supply through the DBL and the volumetric O_2_ consumption/production rate at the sand grain surface from the single sand grain model were used to derive a power-law relationship that can predict the volume of anoxic microenvironments in a given volume of sandy sediment (Fig. S9 and supporting information text). To validate the relationship, we compared the outcomes to experimentally derived denitrification rates which were observed in the presence of O_2_ in sandy sediments from the North Sea^11^. When O_2_ consumption rates, grain sizes and flow velocities from the experiment were used as input parameters, the relationship predicted N_2_ production in anoxic microenvironments to start when pore water O_2_ concentrations dropped below ~ 90 µmol O_2_ L^−1^ (Fig. 4A). The N_2_-concentration increased exponentially until O_2_ dropped to zero, which is in good agreement with the exponential N_2_ production observed experimentally. Overall, the relationship could explain around 74% of the total N_2_ production that was experimentally observed under oxic conditions (220 to 1 µmol O_2_ L^−1^; Fig. 4A and Fig. S10). The other 26% of experimentally determined N_2_ production occurred when pore water O_2_ concentrations were above 90 µmol O_2_ L^−1^, where the relationship predicted that anoxic environments would not yet have started to form. This implies that the low, but linear rates observed under high O_2_ concentrations (above 90 µmol O_2_ L^−1^) are likely derived from microorganisms carrying out denitrification in the presence of O_2_ i.e. so-called aerobic denitrification^8,11,61^.

The power-law relationship was then integrated into a reaction-transport model developed to predict solute (e.g. O_2_ and nitrate) exchange fluxes in permeable sandy sediments^5,13,62^. The modified reaction-transport model revealed that by neglecting anaerobic denitrification in anoxic microenvironments, the N_2_-fluxes from sandy sediments in the German Bight (North Sea) were underestimated by ~ 33% in our previous study^5^. Subsequently, we tested whether anaerobic denitrification in anoxic microenvironments has the potential to contribute to total N_2_ fluxes in other silicate shelf sediments, where O_2_ rates, sand grain size and bottom water velocities differ significantly from the German Bight. We used previously published data from six globally distributed sand sites as input parameters for our modified reaction transport model^4,6,7,11,12,61^. These sites represent a broad range of shelf ecosystems including; coastal upwelling off North-West Africa, a sandy beach seepage face (Sangguo bay, China), a temperate coastal sea (North Sea); a shallow subtropical ocean basin (Gulf of Mexico), a temperate coastal ocean (the South Atlantic bight) and shallow coastal sediments in Australia. All sites were characterized by silicate sands with grain sizes ranging from 102 to 700 μm, bottom water velocities between 0.1 and 0.2 m s^−1^ and denitrification rates from 0.04 to 11.8 µmol N L^−1^ h^−1^.

Despite the large variations in measured environmental conditions and estimated volumetric rates, the upscaling approach indicated that anaerobic denitrification within anoxic microenvironments in oxic sands always contributed substantially to the total denitrification and ranged between 8 and 62% (average 33%; Table S2). The largest source of uncertainty in these estimates is the scaling from the measured bulk rates to the volumetric rates within microbial colonies on sand grains (see supplementary information text). To account for this uncertainty, we varied the estimated volumetric rates in microbial colonies by ± 50%, resulting in an error margin ranging from 4 to 22% (see methods, supplementary information text, and Fig. S14). Our findings imply that anaerobic denitrification in anoxic microenvironments is likely responsible for the previously observed denitrification in oxic sands^6–8,11,17^.

Although our results may not be directly transferable to the ~ 20% of sandy shelf sediments dominated by carbonate sands^63^ or to impermeable sandy sediments (see limitations in the supplementary information text) they indicate that anaerobic microbial activity within anoxic microenvironments is a substantial driver of the N-loss via denitrification that has previously been experimentally observed within the oxic zone of silicate sandy sediments (Fig. 4A, Table S2). The close proximity of oxic and anoxic microenvironments on the surface of silicate sand grains likely also plays a role in the strong coupling of nitrification and denitrification in sandy sediments^5^. Likewise, the presence of anoxic microenvironments might enhance spatial overlaps between other processes such as sulfide oxidation and sulfate reduction^64,65^ as well as influence the mobilization and redistribution of iron and manganese-oxides in silicate sands^36,66,67,68^.

The discovery of anoxic microenvironments on the surface of individual sand grains reveals a heretofore-unknown mechanism that allows anaerobic microbial activity to occur in environments where O_2_ is rapidly replenished by advective pore water flow (Fig. 4B). Our combined results indicate that anaerobic denitrification in these anoxic microenvironments is a substantial sink for anthropogenic-N entering the oceans and thus plays a key role in marine biogeochemical nitrogen cycling.

## Materials and methods

### Sample collection

Sediment samples and sea water for the incubations were collected from an intertidal flat in Dorum, North Sea (53.64° N, 8.49° E) in January 2021. A spatula was used to collect the benthic surface sediment (top 1 cm), collected in a pre-washed plastic transport box, and transferred to the lab in Bremen in a cool box. Sediment was stored immersed in sea water in the dark at 12 °C on shaker and sub-samples were collected for the incubations.

### Microbial colonization and cell counting on sand grains

Silicate sand grains were incubated for 15 min in the dark with SYBR Green I (1:10,000 dilution in DMSO). Subsequently the sediment was washed twice with phosphate-buffered saline (PBS). SYBR Green I stains all the microorganisms containing dsDNA (double-stranded DNA) and is used to visualize the colonization of microorganisms on the sand grain surface. Subsequently, sand grains were placed in a frame (10 × 10 mm; created using transparent tape) on standard glass slides. Fluorescent microscopes (Olympus microscope BX53 and Zeiss Axio Imager M2) were used to image green fluorescence (exc.: 469/35 nm, em.: 510/42 nm) from stained dsDNA and chlorophyll-*a* auto-fluorescence (exc.: 469/35 nm, em.: > 590 nm). The chlorophyll-*a* auto-fluorescence was used to distinguish photosynthetic microorganisms from other organisms^69,70^. Staining and fluorescence z-stack images were used for cell counting on a total 50 sand grains (Fig. 1, S1 and S2). Regions of interest (ROI) were chosen randomly on the sand grain surfaces for counting the number of cells per area.

Subsequently, the cell numbers per sediment volume were calculated by multiplying the cells per area (average 8.57⋅10^5^ cells cm^-^^2^ ± 2.8⋅10^5^ cells cm^−2^) with the surface-to-volume ratio (Eq. 1). For this, we first determined the grain size distribution from images of the collected sand grains (*n* ~ 400, Fig. S11). The images were processed in Matlab (R2019a, Mathworks) to determine diameters (d_g_, Fig. S11). The porosity (θ, ratio of void volume and total volume) was estimated by preparing 10 ml of water saturated sediment, which was left overnight in the oven to dry (Fraser, 1935). From the weight difference the void volume was used to calculate the porosity of 0.4. The surface to volume ratio (S_V, T_) of the sediments was then calculated based on the measured grain size distribution using:

1 $$\:{S}_{V,T}=6\left(1-\:\theta\:\right)\:{\int\:}_{{d}_{2}}^{{d}_{1}}\frac{p\left({d}_{g}\right)}{{d}_{g}}\text{d}{d}_{g}$$

where p(d_g_) is the probability density function of the respective sediment fractions and d_g_ the diameter of the grains. The average surface to volume ratio in our case was calculated to be 153 cm^2^ cm^−3^ resulting in average cell numbers of 1.3⋅10^8^ cells cm^−3^ of sediment.

### Metagenomic analysis of photosynthetic and denitrifying communities

A previously published metagenome^64^ was reanalyzed to investigate the photosynthetic community composition and relative abundance of denitrification genes. The photosynthetic microbial community composition based on SSU rRNA gene sequences in the metagenome was determined using phyloFlash v3.4.1^71^, using the previously outlined approach^72^. Briefly, 16S and 18S rRNA reads were mapped onto reference databases using phyloFlash^71^. For 18S rRNA based analysis, the PR2 version 5.0.0 database was used^73^. For 16S rRNA, a database was created using all bacterial and archaeal entries of the SILVA SSU database version 138^74^ together with the PhytoREF chloroplast 16S rRNA database^75^. Reads assigned to chloroplasts in the 18S rRNA analysis were removed.

To estimate the abundance of denitrifying genes, trimmed reads from the metagenomes were compared against the custom gene reference databases using DIAMOND v2.0.15^76^. To remove false positives, protein sequences from all genomes of species representatives in the genome taxonomy database (GTDB) version R207^77^ and the genomic catalog of Earth’s microbiomes^78^ were used as the outgroup of the genes. The identified reads were searched against the outgroup using DIAMOND v2.0.15 with the same parameters. Reads meeting the following requirements were considered: (1) the match score of the read against the gene reference database > = 100 and (2) the ratio of the match score against the gene database divided by the match score against the outgroup > = 0.9. The read coverage of denitrification genes was normalized by the gene length and the read coverage of the beta subunit of RNA polymerase, RpoB. The obtained fraction represents the estimated proportion of denitrifying microorganisms in the metagenomes, assuming a single gene copy per organism.

### Sensor particle fabrication

Sensor particles were prepared by precipitation as described earlier^79–82^. The sensor particles consist of two dyes, namely, platinum(II)-5,10,15,20-tetrakis-(2,3,4,5,6-penta- fluorophenyl)-porphyrin (PtTFPP; exci.: ~400 nm or ~ 540 nm, em.: ~650 nm) and macrolex fluorescent yellow 10GN (MY; exc.: ~450 nm, em.: ~480 nm). The former is an oxygen (O_2_) dependent dye and the latter is a reference dye to compensate for bleaching of sensor particles and an inhomogeneous illumination^83,84^. Further, MY acts as an antenna dye which allows for the excitation of both dyes with a wavelength of 450 nm. The stock solution of sensor particles was centrifuged for two minutes (Eppendorf centrifuge 5430R) and the supernatant was replaced with collected seawater, this was repeated three times. For calibration, the sensor particle solution was filled in a 6 ml glass vial (Exetainer, LabCo, UK) and O_2_ concentration in the solution was controlled by degassing with N_2_ gas and air (21% O_2_). The O_2_ concentration was monitored using needle optodes (Pyroscience). Correlation between the ratiometric signal and O_2_ concentrations was established using the curve fitting tool in Matlab (see Fig. S12).

### Volumetric O_2_ consumption rate estimates using sensor particles

An acrylic microfluidic chip was designed in which sand grains were coated with sensor particles and incubations were performed (chamber dimensions 35 × 7.8 × 2 mm, Fig. 1D). A thin layer (~ 600 μm) of sand was gently spread out in the chamber of the microfluidic chip using a sterile lab spatula and 1 ml of the prepared sensor particle solution was injected into the chip from the top using a syringe. The added solution was slowly removed from the chip using 1 ml syringes attached at the outlets. This procedure was repeated three times resulting in a homogenous coating of the sediments with sensor particles, keeping the pore space free from sensor particles. The outlets were sealed and the chip was filled with filtered (0.2 μm) seawater, targeting sediment-attached microorganism based on studies indicating that 95 − 99% of microorganisms in silicate continental shelf sediments are found on sand grains rather than in the porewater^43,85,86^. The top of the microfluidic chip was covered using a glass coverslip. The incubation was performed under low light conditions of around 0.4 µmol photons m^2^ s^−1^, which is in the lower range of light reaching intertidal or subtidal shelf systems (e.g^44,87,88^. Although we could not measure the light directly within the chip it is likely that the light levels within the chip were higher due to increased irradiance (caused due to small and confined space)^89^.

The prepared microfluidic chip was mounted on an inverted microscope (Leica DMI 6000B) with a sCMOS camera attached (pco.panda 4.2). An LED light source (Omicron LedHUB^®^ – High-Power LED Light Engine) with a wavelength of 455 nm and output power 2400 mW was used for excitation. Images were recorded through the bottom glass with an exposure of ~ 200 ms and stored using the pco.camware 4.11 software. A polyester filter (Lee filter-101 Yellow; emi.: >480 nm) was applied to ensure that only emissions from the sensor particles were recorded. During the incubation, images were captured approximately every 20–30 min until no substantial changes were observed, i.e. the microfluidic chip turned anoxic. RGB images were captured at a resolution of 2048 × 2048 pixels. After incubations, the density of sensor particles was quantified from scanning electron micrographs as 5 particles per µm^2^ (Fig. S13 A-B). Additionally, lifetime imaging using a pco.1600 camera (Excelitas PCO AG) attached to a custom-made modulator for light source triggering was used to capture lifetime images from the sensor particles. The lifetime imaging confirmed the homogeneous distribution of the sensor particles (Fig. S13 C-D).

All post-processing was carried out using Matlab (R2019a, Mathworks). A ratiometric imaging approach was used to estimate the volumetric O_2_ consumption and production rates in the acquired RGB images. For each time point 10 images were captured and averaged, the raw images were split into red and green channels and the ratio was calculated:

2 $$\:Ratio\:=\frac{\:Red\:channel\:\left(indicator\:dye\right)}{Green\:channel\:\left(reference\:dye\right)}$$

The ratio is related to the specific O_2_ concentration through the Stern-Volmer equation (see Fig. S12). The O_2_ production and consumption rates from the microfluidic incubation were calculated by estimating the pixel wise differences of the ratio for two time points in the chip, divided by the incubation time:

3 $$\:R=\:\frac{f\left({Ratio}_{t1}\right)-{f(Ratio}_{t0})}{t}$$

R is the O_2_ rate (µmol O_2_ L^−1^ h^−1^), Ratio_t1_ is ratiometric values at time t_1_, Ratio_t0_ is the ratiometric values at time t_0_, t is incubation time in hours (t_1_ - t_0_). In the approach, we assume that at t_0_, the O_2_ concentration is homogeneously distributed around the sand grain. The O_2_ respiration rates calculated using this approach for the entire incubation period of four hours are rather a conservative estimate, due to no-flow in the microfluidic chip causing diffusion limitations. For processing of the O_2_ rate matrix, a threshold was set to remove outliers, and data was smoothed using the medfilt2 function with a 5 × 5 kernel. O_2_ rates were masked through thresholding based on the chlorophyll-*a* auto-fluorescence. Thereby, net consuming microbial colonies are separated from net producing microbial colonies for subsequent quantifications. Note that in some cases O_2_ production was observed in areas containing non-autofluorescent microorganisms. These regions were not abundant and are likely due to the integration of emission signals from the sand grains due to their 3D structure and reflections.

### Modeling single sand grains and the formation of microenvironments

To investigate the effect of the patchy microbial colonization on a single sand grain under varying environmental conditions, we developed a two-dimensional multiphysics model (Comsol multiphysics 5.6). The model domain consists of circular sand grain with a reactive domain (5 μm) on the sand surface (Fig. S7). The flow field is calculated by solving the laminar Stokes equations:

4 $$\:0=\:-\:\nabla\:\text{p}+\varvec{\mathbf\upmu}\:{\nabla\:}^{2}\varvec{u}$$

5 $$\:\nabla\:\cdot\:\varvec{u}=0$$

**u** is the velocity vector, p the pressure, µ dynamic viscosity (1.22⋅10^−3^ Pa⋅s) and ∇ the gradient-operator.

The oxygen (O_2_) and dinitrogen (N_2_) distribution was calculated by solving the stationary advection-diffusion equations:

6 $$\:0=D\:{\nabla\:}^{2}{C}_{O2,N2}-\varvec{u}\cdot\:\:\nabla\:{C}_{O2,N2}-{R}_{c}\:\:$$

D is diffusion coefficient (1.1⋅10^−9^ m^2^ s^−1^ for O_2_, and 2.1⋅10^−9^ m^2^ s^−1^ for N_2_ at 25 °C corrected for EPS in the colonization patches^19^, ∇ the gradient-operator, C_O2_ is concentration of O_2_ and C_N2_ the concentration of N_2_, **u** is the velocity vector. No-flux, slip boundary conditions were applied at the top, and bottom boundaries and no-slip boundary conditions on the sand grain boundary (see Fig. S7). Inlet and outlet boundaries as inflow and outflow for the assigned pore water flow. For R_c_, we applied a sinusoidal function along the surface of the sand grain to produce alternating patterns of production and consumption in a 5 μm thin layer:

7 $$\:{R}_{c=\:}\left({R}_{O2,N2}\right)\cdot\:(\text{sin}\left(\text{atan}\left(\frac{y}{x}\right)\cdot\:a\right))\:\cdot\:{f}_{O2,N2}(\text{C})$$

Where y and x are the spatial coordinates. R_O2, N2_ are the volumetric O_2_ consumption rates within microbial colonies at the sand grain surface. These colonies and their associated O_2_ consumption rate occur in 1/1000 of the entire pore volume. To determine R_O2, N2_, we used our measured O_2_ consumption rates that represent the volumetric rate of the entire pore volume. To convert the measured rate from the entire pore volume to the microbial colonies, we divided the 25th percentile of measured rates (-46 µmol O_2_ L^−1^ h^−1^) by the cell numbers of 1.3 ⋅10^8^ cells cm^−3^ to estimate the cell-specific rate of -0.4 fmol O_2_ cell^−1^ h^−1^. Subsequently we divided the cell-specific rate by their characteristic biovolume (0.4 µm^3^ which yields the rate of the microbial colonies of R_O2_ = -955 mmol O_2_ L^−1^ h^−1^. Phototrophic microorganisms sparsely colonized the surfaces of sand grains, resulting in O_2_ production occurring on approximately one-third of the sand grain surfaces, while O_2_ consumption takes place on approximately two-thirds of the sand grain surfaces (Fig. 2D). To take this into account, the sinus curve (R_c_) was shifted downwards, so that the final colony sizes are 76 μm for the net O_2_ consuming microbial colonies and 38 μm for the net O_2_ producing microbial colonies. Four consuming and four producing colonies were modelled along the sand grain (a = 4). The final minima of R_c_ are at R_c, min_ = -955 mmol O_2_ L^−1^ h^−1^ representing net-consuming colonies and the maxima of R_c_ are at R_c, max_= 514 mmol O_2_ L^−1^ h^−1^ representing net producing colonies. When considering the lower cell numbers of 3.5 ⋅10^6^ cells cm^−3^ of phototrophic organisms the net producing colony values correspond to 7.1 fmol O_2_ cell^−1^ h^−1^. Employing this approach, we assume that within the microbial colonies, the volumetric rates follow a normal distribution, gradually decreasing from the core towards the outer regions.

Further, for the consumption of O_2_, we incorporated a Michaelis-Menten kinetic: f_O2_(C) = C_O2_/(K_m_+C_O2_), where C_O2_ is the concentration of O_2_, K_m_ is the half-saturation coefficient of the O_2_ consumption rate (K_m_= 0.1 µmol L^−1^). The production of N through denitrification was set proportional to the volumetric O_2_ consumption rate at a ratio of 10:1, which is based on empirical observations (See Fig. S14) with an inhibition constant of N at C_inh_ = 0.1 µmol L^−1^. The inhibition was incorporated through the function f_N_(C) = C_inh_/(C_inh_+C_O2_) (see also^90^.

We first performed 160 model runs, of which, 80 runs involved the direct application of Eq. 7, with variations in porewater velocities ranging from 0 to 500 μm s^−1^ and pore water O₂ concentrations ranging from 0 to 100 µmol L^−1^. Subsequently, a second round of model runs was conducted in which values within the reactive domain with R_c_ > 0 were set to zero. The parametrization for porewater velocities and pore water O_2_ concentrations was adjusted to the same values as in the first run with consumption and production. To evaluate the sensitivity of the parameters utilized in the model, we ran in total 1764 model runs expanding the range of applied parameters to conditions typically present in other sandy sediments of other regions (see supplementary information text).

### Estimation of the sand grain roughness

To quantify sand grain surface roughness and evaluate its impact on the diffusive boundary layer (DBL), we followed an approach based on Yao & Li (2023)^91^, adapted to a 2D framework using microscopic images. High-resolution images of individual sand grains (*n* ≈ 40) were analyzed to extract their outlines, which were then compared to their respective convex hulls (Fig. S16). Surface roughness (in micrometers) was calculated as the radial difference between the actual grain outline and its convex hull, providing a geometric measure of the surface irregularity. The processing was performed in Matlab R2019b (Mathworks).

### Relationships for the development of anoxic microenvironments

To incorporate our model results into a previously published reaction-transport model, we derived a non-dimensional number: “Sand_DBL_”. The Sand_DBL_ number represents the ratio of diffusive exchange of O_2_ into the boundary layer and the activity of the microbial colonies. First, the diffusive boundary layer thickness δ around a single sand grain was estimated by applying a previously derived scaling law^92,93^:

8 $$\:{\updelta\:}=\:\frac{r}{Sh}$$

where *r* is median radius of the sediments, *Sh* is Sherwood number Sh = 1 + 0.62・Re^0.41^ Sc^0.33^, *Re* is Reynolds number Re = Ur/*v*, *U* is pore water velocity, *v* is kinetic viscosity, *Sc* is Schmidt number Sc = *v*/D, *D* is diffusion coefficient of O_2_ in water. The Sand_DBL_ number (analog to the Damköhler number) is then calculated by estimating the diffusive time scale to the reaction time scale:

9 $${\text{Sand}}_{{{\text{DBL}}}} = \frac{{\updelta ^{2} }}{D} \cdot \frac{{R_{{O2}} }}{{C_{0} }}$$

where *R*_*O2*_ is reaction rate, C_0_ is bulk O_2_ concentrations. We found that for Sand_DBL_ < 10 microbial colonies on the sand grain are mostly oxic and for Sand_DBL _>1000 the microbial colonies are mostly anoxic. For the intermediate range 10 < Sand_DBL_ < 1000 we estimated the best fit between anoxic volumes of the microbial colonies and the Sand_DBL_ (for additional details see supplementary information text). A total of 1764 model runs were used for the determine the relationship between the Sand_DBL_ number and the volume of anoxic microenvironments (Supp. Equation 1 and Supp. Equation 2).

### Areal denitrification estimates

We used a model approach to estimate the contribution of denitrification within anoxic microenvironments to total denitrification based on field-obtained data. The model allows an effective mixing depth *D* in sandy sediments to be calculated as a function of time *t*^5,13,62^:

10 $$\:\:D=\:\frac{1}{k\:\text{ln}(\frac{0.42\:{k}^{2}K\:{h}_{m}t}{{\uptheta\:}}+1)}$$

Where h_m_ is the hydraulic head (m), θ the porosity, k the wavenumber λ/(2·pi), λ the characteristic bedform size, K is the hydraulic conductivity (m s^−1^). The bedform size is determined following the empirical relationship λ = 490⋅d_g_^13^. The hydraulic head follows h_m_ = 1000⋅U^2^⋅0.1/10000, where U is the bottom water velocity (as in^94^. The hydraulic conductivity is calculated as K = P / ν ⋅ g, where P is the permeability which can be determined by the grain size: *p* = 9.869⋅10^−13^⋅735⋅d_g_^2^⋅1e^−6^^95^, ν is the temperature-dependent kinematic viscosity and g the acceleration by gravity. To determine the characteristic pore water velocity U, we used the equation U = k⋅K⋅h_m_^62^. To take anoxic microenvironments into account, we calculated the O_2_ penetration depths by estimating the time until O_2_ is depleted: t = c_O2_ / R, where c_O2_ is the temperature dependent bottom water concentration of O_2_. This timescale t is then used in Eq. 10. Subsequently, we mathematically split the oxic sediment into four layers and in each of these layers we calculated the Sand_DBL_ number to determine the volume of anoxic microenvironments (Supp. Equation 3). We then multiplied the volume of anoxic microenvironments with the volumetric denitrification rate. Integrating the resulting values over the O_2_ penetration depth yields the denitrification flux in anoxic microenvironments. To determine denitrification fluxes from the anoxic sediment, we calculated the nitrate penetration depth by calculating the time until nitrate is depleted and by using this time in Eq. 10. Finally, we integrated the volumetric denitrification rate over the nitrate penetration depth to estimate the denitrification flux below the oxic-anoxic interface.

For the parametrization we compiled a dataset with globally distributed grain sizes, O_2_ respiration rates, denitrification rates, bottom water velocities, bottom water O_2_ concentrations and bottom water nitrate concentrations (Table S2). For the implementation of the parameterization to field data from other published studies, we used the reported O₂ bottom water concentrations and temperatures from the respective publications and adjusted for temperature-dependent solubility, diffusion coefficients, and kinematic viscosity accordingly. In case if the specific data was not provided in the publications, we calculated O₂ solubility based on the study site’s temperature and salinity. Additionally, the O₂ diffusion coefficient, which is temperature-dependent, was adjusted according to the temperature values reported in the respective publications by the authors. When using this dataset for the parameterization, we found the contribution of denitrification in anoxic microenvironments to range between 8 and 62%. Sensitivity testing of key parameters influencing Sand_DBL_ and the power-law for prediction of anoxic microenvironments revealed that the volumetric rates within microbial colonies were the most sensitive parameter. In our upscaling approach, we estimated these volumetric rates on sand grain surfaces by scaling them relative to the bulk volumetric rates reported by the authors. We accounted for the uncertainty of this approach by varying the resulting volumetric rates within microbial colonies by +- 50%. The resulting range is provided as the uncertainty in the estimated contributions of anoxic microenvironments to total denitrification (see Table S2). The Matlab (R2019a, Mathworks) script for the calculations can be found on github (https://github.com/SoerenAhmerkamp/SandDBL).

## Electronic supplementary material

Below is the link to the electronic supplementary material.

Supplementary Material 1

## Data Availability

The MATLAB (R2019a, MathWorks) code and the COMSOL Multiphysics 5.6 model is available online at https://github.com/SoerenAhmerkamp/SandDBL. All measurement data are included within the manuscript or provided in the Supplementary Material.
