# Supplementary material for: Microenvironments on individual sand grains enhance nitrogen loss in coastal sediments
Source: Sci Rep. 2025 May 11;15:16384. doi: 10.1038/s41598-025-00755-3 (PMC12066709; doi:10.1038/s41598-025-00755-3)
Supplement: Supplementary file 1 — Supplementary Material 1 [file 41598_2025_755_MOESM1_ESM.docx]

**Supporting Information**

Microenvironments on individual sand grains enhance nitrogen loss in coastal sediments

Farooq Moin Jalaluddin^1^, Soeren Ahmerkamp^1,2^, Hannah K. Marchant^1,3^, Volker Meyer^1^, Klaus Koren^4^, Marcel M. M. Kuypers^1^

**Affiliations** ^1^ Max Planck Institute for Marine Microbiology, 28359 Bremen, Germany
 ^2^ Leibniz Institute for Baltic Sea Research Warnemünde, Rostock, 18119, Germany
 ^3^ MARUM – Center for Marine Environmental Sciences, University of Bremen, 28359 Bremen, Germany
 ^4^ Aarhus University Centre for Water Technology, Department of Biology, Aarhus University, 8000 Aarhus, Denmark

Corresponding author: Soeren Ahmerkamp
 **Email**: [sahmerka@mpi-bremen.de](mailto:sahmerka@mpi-bremen.de)

**This PDF file includes:**

Supporting text

Figures S1 to S16

Tables S1 and S2

SI References

**Supporting Information Text**

**Extended description of anoxic microenvironment formation in the single-sand grain model and derivation of empirical relationships for anoxic microenvironment formation**

Our single sand grain model shows that the volume of anoxic microenvironments correlates with the ratio of diffusive O₂ supply to the volumetric reaction rate at the grain surface. This ratio is captured by the non-dimensional "Sand_DBL_" number (see Methods). To derive power-laws for anoxic microenvironments based on the SandDBL number, 1764 model runs were carried out, out of which 882 model runs included both O_2_ consumption and O_2_ production and 882 model runs included only O_2_ consumption. These model runs showed that below a Sand_DBL_ of 10 the sand gain surface is oxic and Sand_DBL_ above 1000 the sand grain surface is fully anoxic. Between 10 and 30 the relationship between Sand_DBL_ and the percentage of microenvironments can be described by

Vol_anoxic_ = 0.043⋅Sand_DBL_^2.05^ (Supp. Eq. 1)

R-squared = 0.8 (consumption and production)

R-squared = 0.6 (only consumption)

Above 30 and below 1000 the relationship between Sand_DBL_ and the percentage of microenvironments can be described by

Vol_anoxic_ =25.04⋅Sand_DBL_^0.20^. (Supp. Eq 2)

R-squared = 0.5 (consumption and production)

R-squared = 0.6 (only consumption)

We found that the absence of photosynthetic O_2_ production slightly changed the R-square value of the power-law but had no significant impact on the power-law coefficients (Fig S9). By incorporating these Sand_DBL_ based power-laws into a reaction–transport model, we can estimate the contribution of reactions within anoxic microenvironments to overall solute fluxes (e.g., nitrate, N₂, O₂) based on bulk sediment measurements, such as O₂ concentrations and pore water velocity (Fig. 3C, Fig. S9, see Methods).

**Sensitivity testing of the single sand grain model**

A mechanistic modeling approach was used to study mass transfer around a single sand grain. The initial parameterization was based on the experimental conditions (see Table S1 for a full overview). To test the validity of the non-dimensional number, we varied the input parameters in a total of 1764 model runs. In these runs we varied the O_2_ consumption rates in the microbial colonies from 300 to 1500 mol m^-3^ h^-1^ and the flow velocity from 1 to 500 µm s^-1^. Additionally, we varied the diffusion coefficients in the pore space from 1.8⋅10^-9^ to 3.0⋅10^-9^ m^2^ s^-1^ (also for the microbial colonies) to account for potential temperature variations between 15 to 35°C. Previously the diffusion coefficient for pore space was set to 2.0⋅10^-9^ m^2^ s^-1^ and for the colonies to 1.1⋅10^-9^ m^2^ s^-1^ (see Fig. S7 and S14). While there is some scattering of data around the presented power-laws, the fitting is generally robust as indicated by the R^2^ of 0.5-0.6 (Fig. S9).

The parameter variations reveal that the O_2_ consumption rate of microbial colonies is the most sensitive parameter in determining the Sand_DBL_ number and in predicting the anoxic microenvironments. When the O_2_ consumption rate was reduced by half, the anoxic volumes decreased, on average, across different inflow O_2_ concentrations from 60% to approximately 40% (Fig. S14 A and B). This effect is more pronounced at high flow velocities (100 µm s^-1^), where the average volume of anoxic microenvironments reduces from 60% to approximately 30%. Given that this factor has the most substantial impact, we subsequently considered a wide error margin of ±50% for the O_2_ consumption within the microbial colonies in the upscaling analysis. This ensures that we capture the potential effects of both increased and decreased activity of microbial colonies on the extent of anoxic microenvironments.

The variation in the diffusion coefficient had minimal impact on the volume of anoxic microenvironments at low O_2_ inflow concentrations (<10 µmol L^-1^). For higher inflow O_2_ concentrations (>10 µmol L^-1^), there was an average decrease of around 20%, for both low and high pore water velocity. Overall, increasing the temperature from 15°C to 35°C resulted in a slight decrease of approximately 6% in the volume of anoxic microenvironments (Fig. S14 C and D), showing the formation of microenvironments in nearly all scenarios.

Further, in our model, we assume that microbial respiration takes place primarily on the sand grains. This assumption is based on the fact that 95% to 99% of microorganisms in silicate continental shelf sediments reside on the sand grains rather than in the porewater^1–3^. To test the impact of a potential additional respiration in the porespace, microbial respiration was varied between the sand grain surface and the pore space. The ratios examined include 100% colonization on the sand grain surface, 99% on the surface with 1% in the pore space, 90% on the surface with 10% in the pore space, and an even distribution of 50% on the surface and 50% in the pore space (Fig. S15). The simulations revealed that for the most realistic scenarios (> 90% : 10%) colonization, the formation of anoxic microenvironments was not affected by the O_2_ respiration in the porespace. A substantial effect of the O_2_ concentration in the porespace was only observed at a ratio of 50%:50% (Fig. S15 D). In this case, the heterogeneity observed on the surface of the sand grain is also visible in the pore space, and the occurrence of anoxic microenvironments on the sand grain surface is amplified by approximately 5%.

Microfluidic experiments were performed under low light conditions to capture both O_2_ production and consumption from the microbial community attached to the sand grains. As only the first few millimeters of silicate sands are exposed to light in situ, we compared the formation of anoxic microenvironments for illuminated and non-illuminated silicate sands in different model runs: i) sediment grains colonized by active primary producers and O_2_ consumers (illuminated sands), and ii) sediment grains where only O_2_ consumers were active (non-illuminated sands). Comparison of the two scenarios revealed that O_2_ production had only a minor impact on the development of anoxic microenvironments, inducing slightly stronger variabilities of O_2_ on the surface of the sediment grain (see Fig. S14). Even though we had O_2_ production and consumption in the experimental set-up, we observed that combined experimental and model results are applicable to both illuminated and non-illuminated silicate sands.

**Limitations of the methods**

The microfluidic chamber experiments performed here focused on measuring the rates of O_2_ consumption and production, as well as assessing the variability of these rates across the surface of individual sand grains. In the current setup in order to observe changes in O_2_ concentrations we had to seal the microfluidic chamber to prevent flow. Introducing flow into the experiment presents challenges, as it could dislodge sensor particles from the sand grain surface and potentially cause sand grains to shift. Moreover, initial tests suggest that flow can lead to clogging of the pore space by sensor particles, creating distortions in the O_2_ measurements. Therefore, conducting experiments to compare the change in flow and boundary conditions using the microfluidic chamber experiments under flow conditions is not feasible with our current setup and falls outside the scope of this study.

The primary distinction between flow and no-flow conditions lies in the alteration of mass transfer towards sand-grain surface when the volumetric rates within microbial colonies become diffusion-limited. This is apparent in the non-linear decrease in O_2_ concentration measurements after one hour, which suggests a diffusion limitation (refer to Fig. S6). Therefore, because we did not apply flow, the O_2_ consumption rates calculated for the entire incubation period should be regarded as conservative estimates. Thus, the lack of flow might impact the overall rates that we measured, but not the observed heterogeneity across sand grain surfaces or the observed formation of microenvironments. In regards to the extent of microenvironment formation; as shown in extended modelling runs (see Fig. S14 A and B), if we apply a higher O_2_ consumption rate in the microbial colonies, then the volume of anoxic microenvironments actually becomes greater.

The mechanistic model provides insight into the formation of anoxic microenvironments around individual sand grains under varying flow and O_2_ concentrations. It is important to note that the model is applicable only to permeable sediments with permeabilities greater than 10^-12^ m^2^, typically corresponding to grain sizes exceeding approximately 100 µm in low-mud sediments, or over 200 µm when mud content is high (see, e.g., ^4^). Further, the model does not fully capture all the factors that contribute to heterogeneous O_2_ concentrations within pore spaces under natural conditions. A key factor is pore space clogging due to extensive biofilm growth^5–8^, aggregate trapping^9,10^, streamer formation^11,12^, tortuosity effects^13,14^. Pore space clogging restricts both pore water flow and O_2_ supply to the microbial community on the sand grains. Sensitivity tests showed that as pore water velocity decreases tenfold from 100 µm s^-1^, the volume of anoxic microenvironments is increased from approximately 30% to 60%. Accurately estimating the impact of pore space clogging on the scale of shelf systems remains a task for future research.

**Supporting Information Figure**


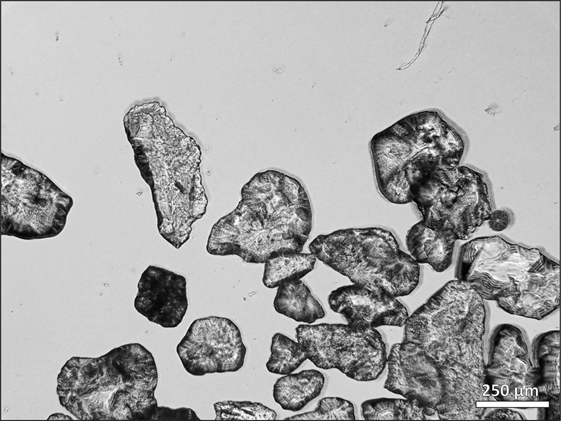


**Fig. S1.** **Photomicrograph of sand grains.** Monochrome bright-field image of the investigated intertidal sand grains reveal heterogeneous shapes and irregularities on the sand surface.


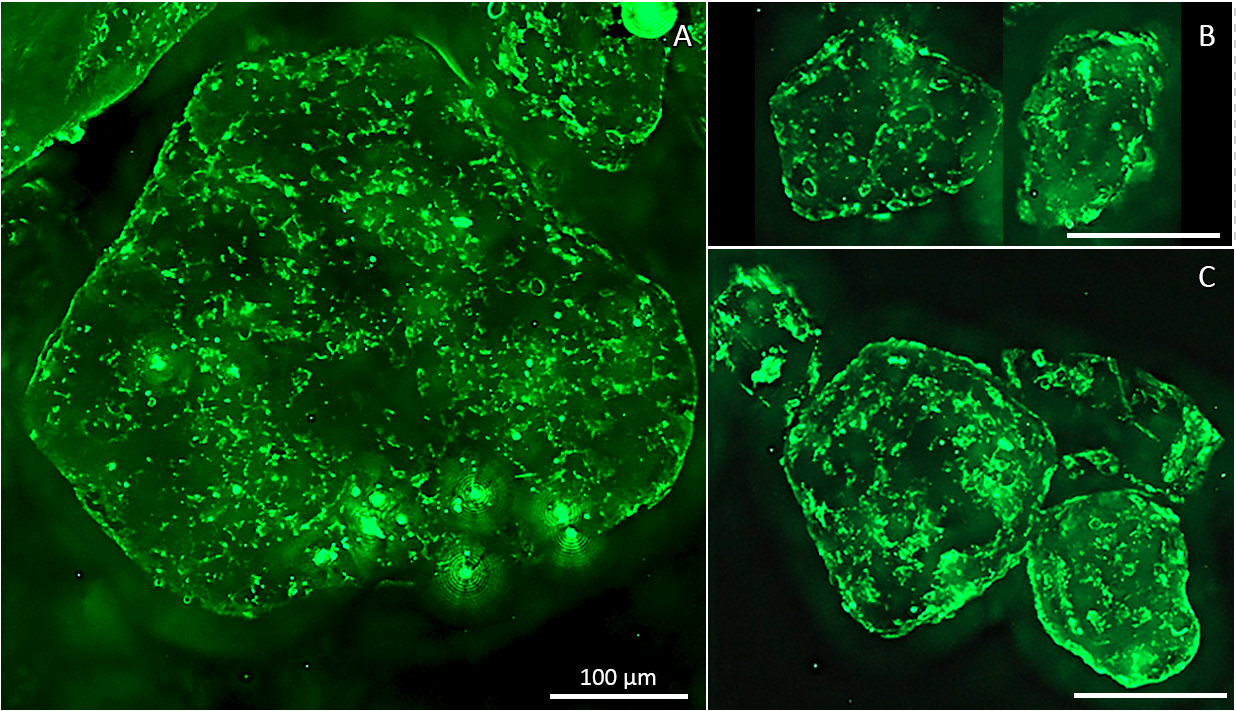


**Fig. S2.** **Colonization of sand grains by microorganisms.** **A-C** dsDNA staining (SYBR Green I, green, exc.: 469/35 nm, em.: 510/42 nm) of representative sand grains showing a patchy microbial colonization. In total 50 sand grains were analyzed for cell-counts. The image represents a maximum intensity projection which was carried out for a z-stack of 25 images.


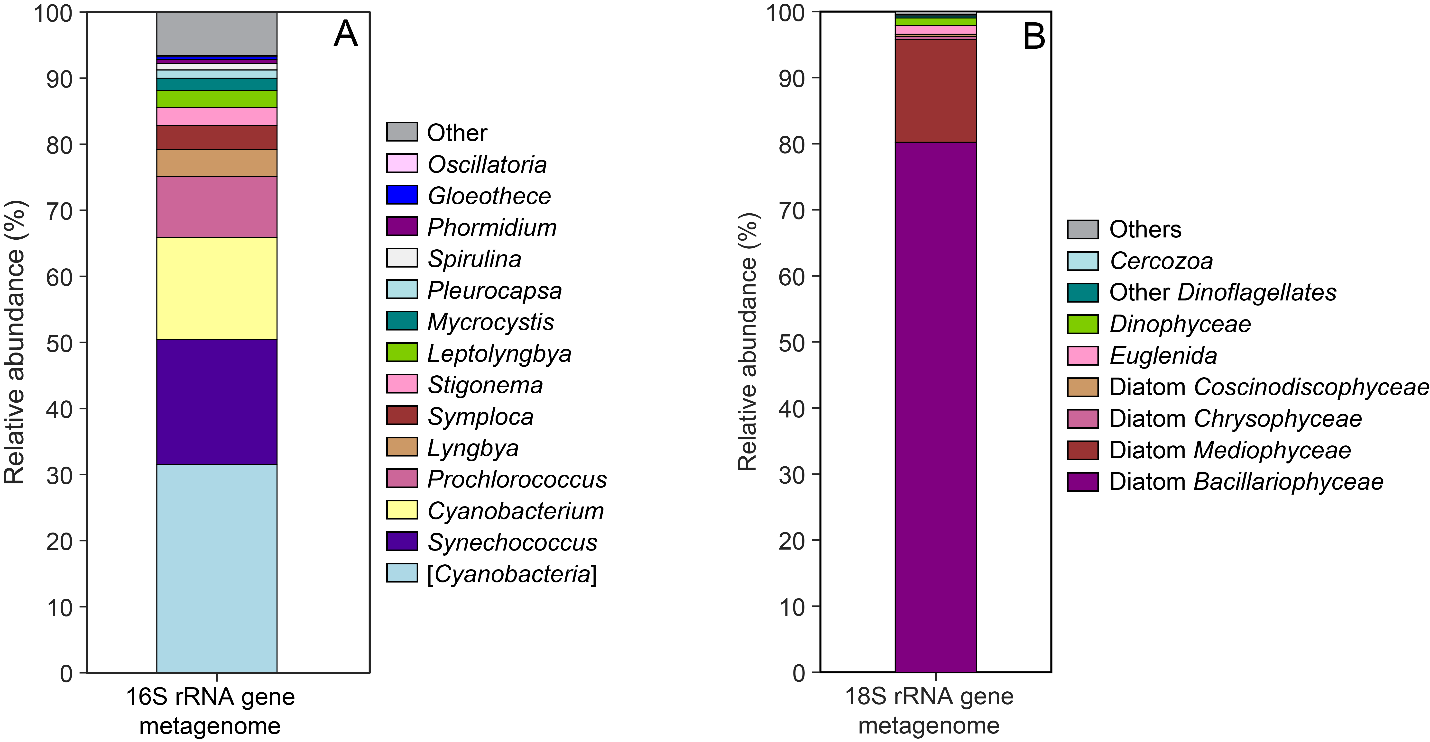


**Fig. S3:** **Relative abundance of photosynthetic microorganisms in an intertidal North Sea sandflat based on taxonomic identity**. **A** *Cyanobacteria* community composition based on 16S SSU rRNA gene reads identified within the metagenome and classified to genus level. Note that [*Cyanobacteria*] refers to sequences which could not be classified below phyla level. **B** Eukaryotic photosynthetic community composition based on 18S SSU rRNA reads identified within the metagenome and classified to order level.


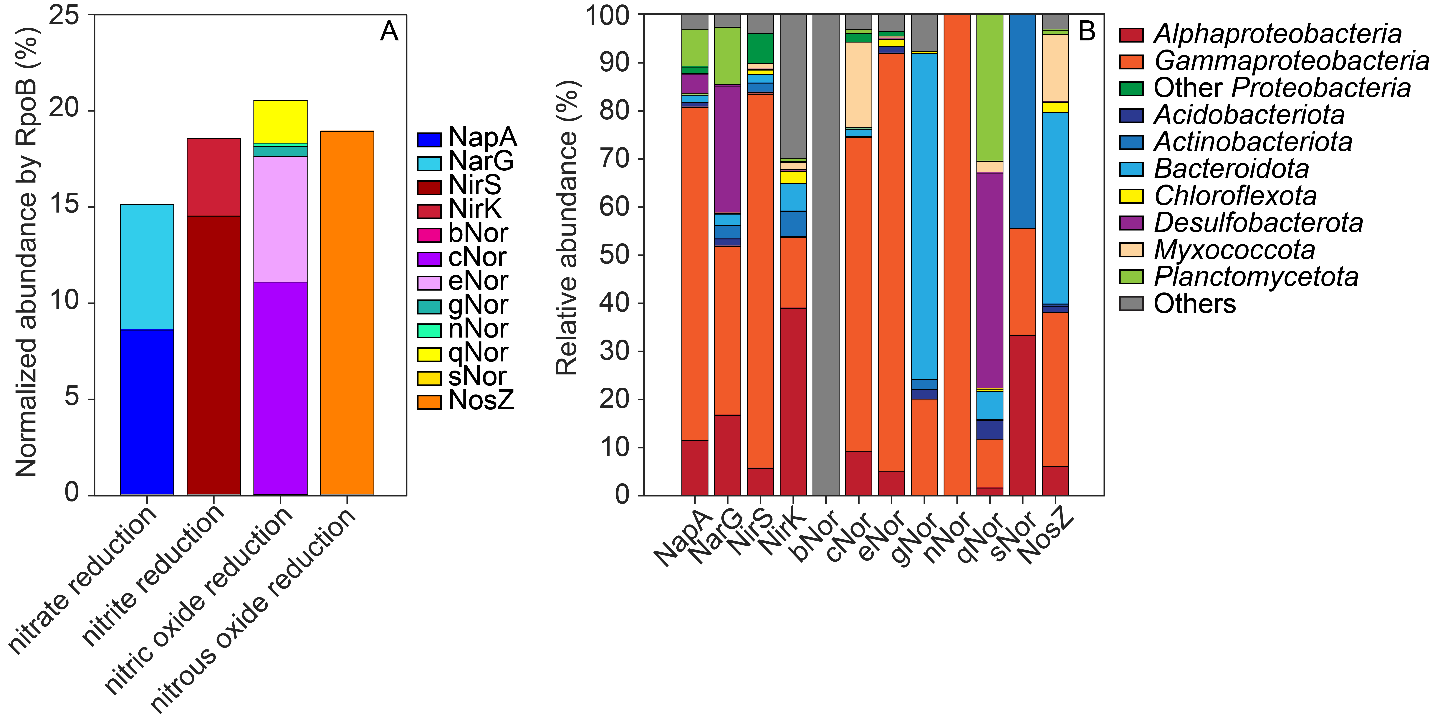


**Fig. S4:** **Phylogenetic affiliation of functional gene reads associated with the denitrification pathway in a metagenome from an intertidal North Sea sandflat. A** Normalized abundance of marker genes associated with nitrate reduction (NarG, NapA), nitrite reduction (NirS, NirK), nitric oxide reduction (bNor, cNor, eNor, gNor, nNor, qNor, sNor) and nitrous oxide reduction (NosZ). **B** Taxonomic distribution of detected denitrification functional gene reads.


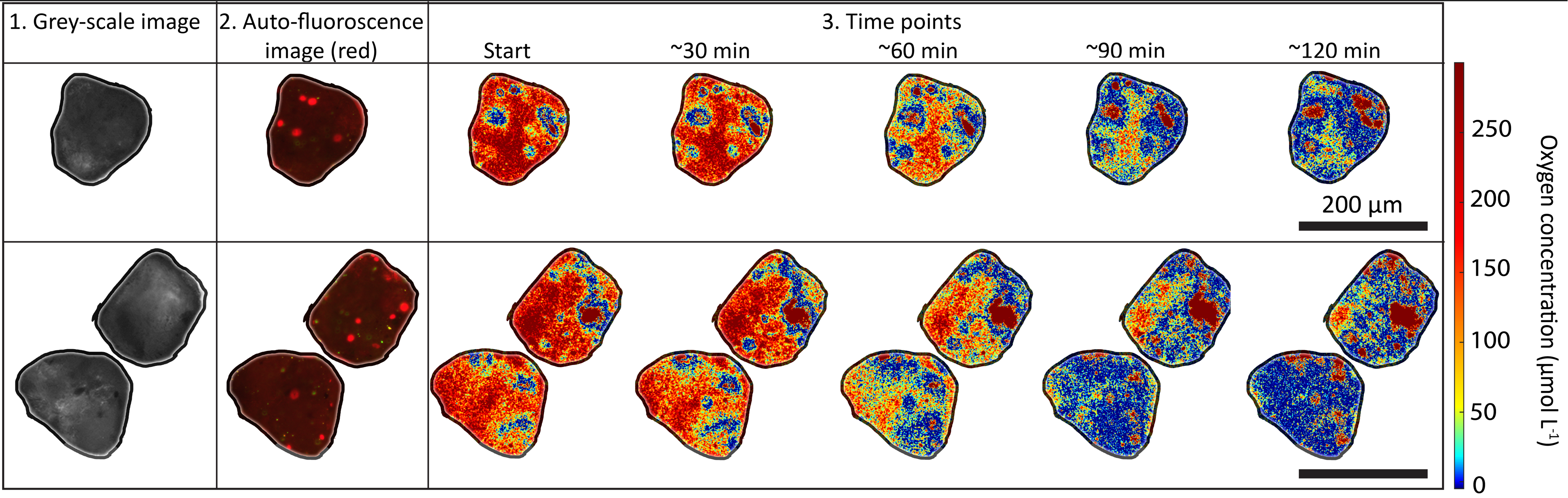


**Fig. S5.** **Formation of anoxic microenvironments on the surface of individual sand grains**. The first column displays grayscale images, while the second column shows auto-fluorescence (exc.: 469/35 nm, em.: > 590 nm) which is indicative of chlorophyll-*a* containing photosynthetic microorganisms. Column three shows the heterogeneous O_2_ concentrations at the surface of sand grains over time.


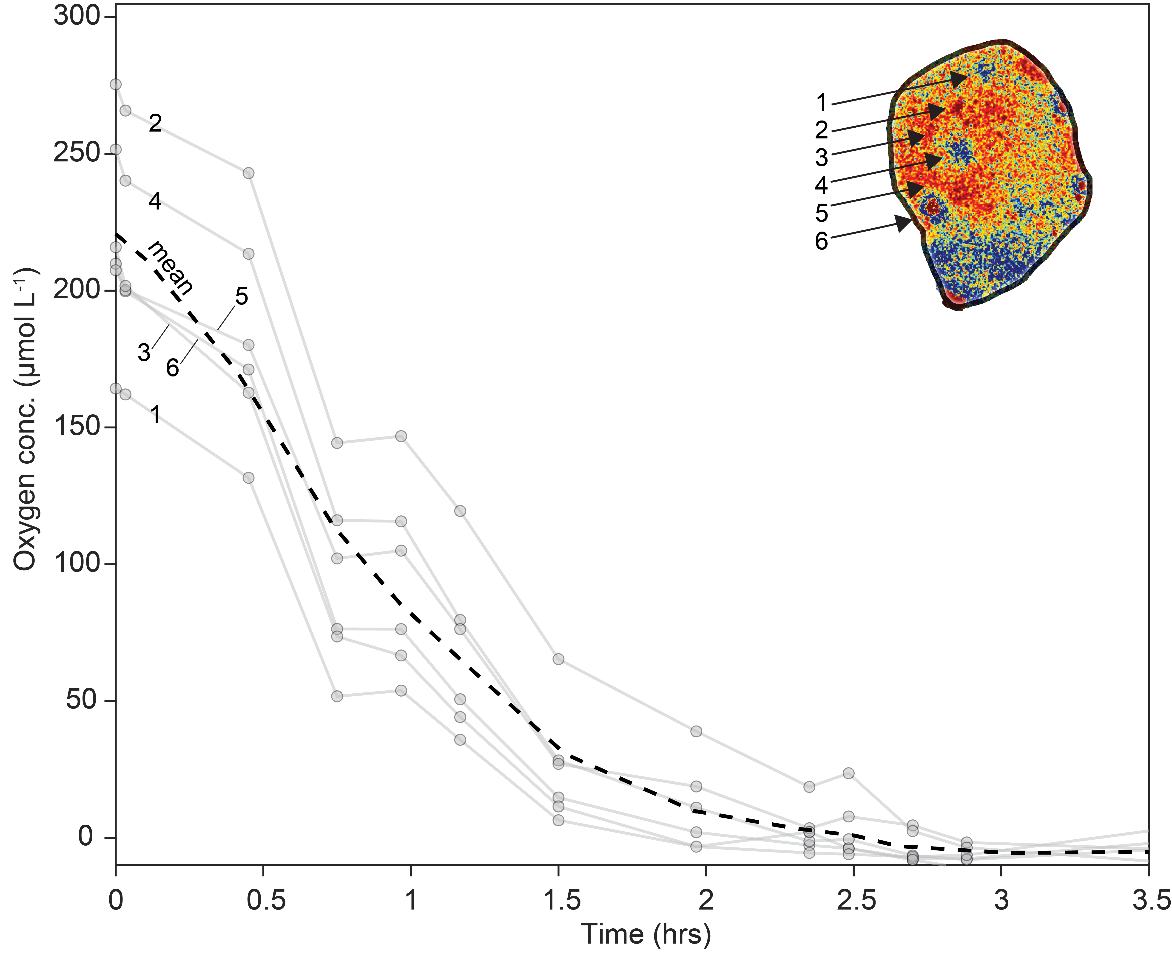


**Fig. S6**. **Changes of the O_2_ concentration on the silicate sand grain surface for randomly selected patches as indicated on the insert.** Grey-lines represent the changes in O_2_ concentration for five different spots and the dashed-line indicates the mean low-pass filtered values for O_2_ concentrations over time. This non-linear decrease suggests that diffusion limitation is impacting O_2_ respiration by limiting O_2_ respiration rates. Thus, the O_2_ consumption rates (which were calculated over the entire incubation period (4h)) should be regarded as conservative estimates.


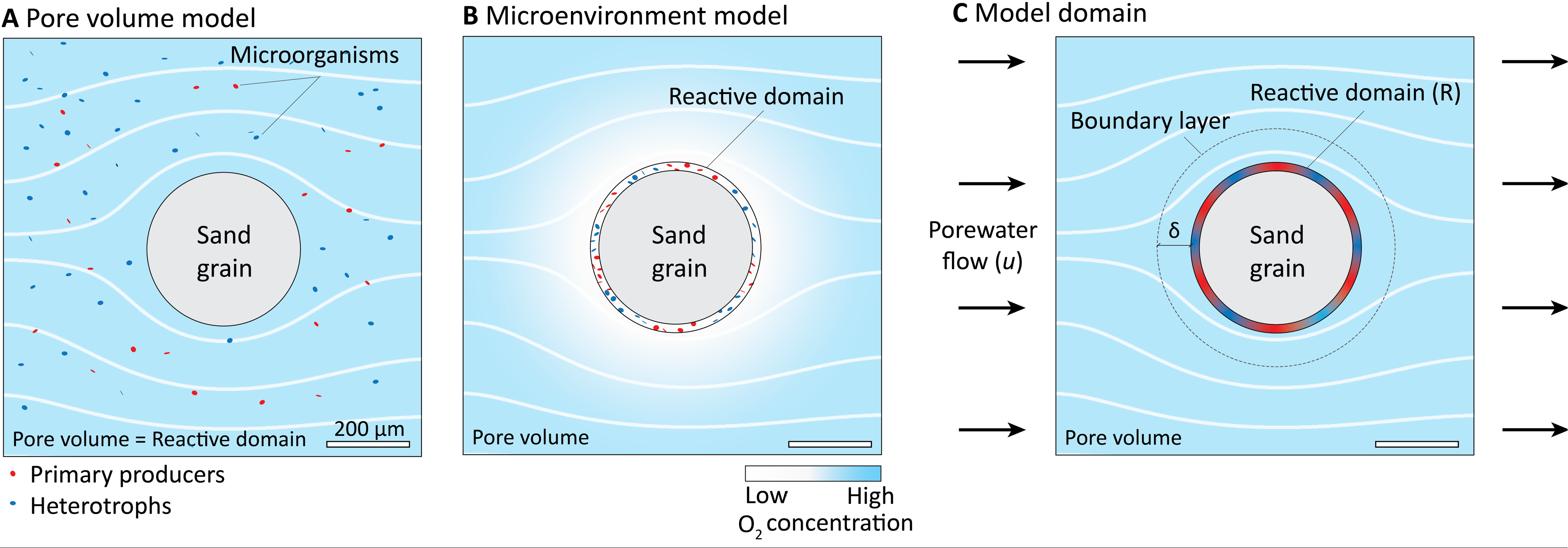


**Fig. S7. A** Schematic of a model that assumes a constant volumetric rate in the pore space, which is representative of a homogeneous colonization by microorganisms. Such models do not differentiate between microorganisms colonizing the sand grain surface and the pore space. **B** Schematic of a model in which the microorganisms only colonize the surface of sand grains which is similar to our observations. **C** Our model domain in which patches of O_2_ consumption and production are modelled to occur within a thin 5 μm layer along the surface of a single sand grain. Blue indicates consumption and red indicates the production of O_2_ (parameters are described in the methods section “Modeling single sand grains and the formation of microenvironments” and “Relationships for the development of anoxic microenvironments”).


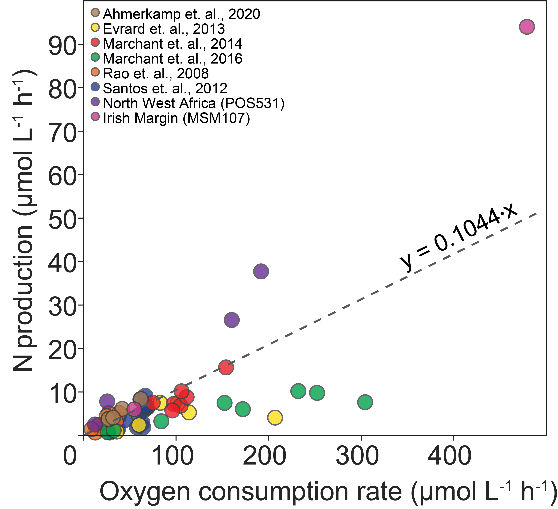


**Fig. S8:** **Correlation between oxygen respiration rates and denitrification rates in sandy sediments**. The dotted line represents a linear regression (y = 0.1044·x, R^2^ = 0.67), indicating a positive relationship between aerobic and anaerobic processes. Data was compiled from multiple studies^15–21^. North West Africa (POS531) and Irish Margin (MSM107) are unpublished data from research expeditions to North West African Shelf and the continental shelf at the Irish Margin, respectively. Previously published dataset adapted and updated^18^.


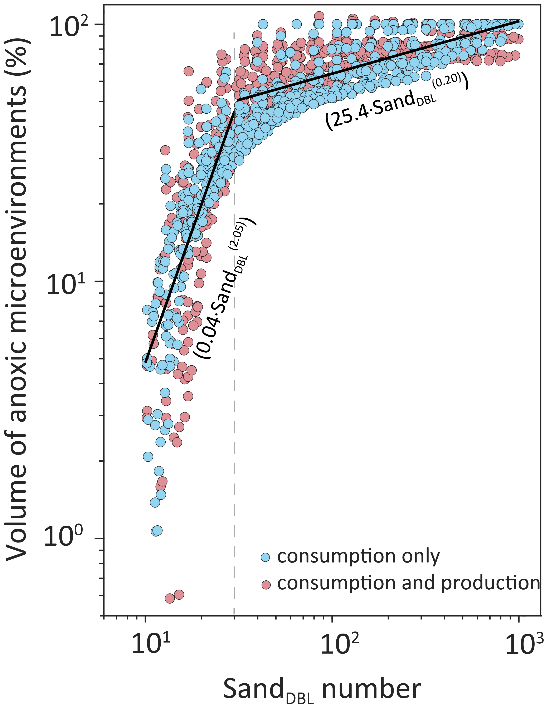


**Fig. S9.** **Power-law of the anoxic volumes based on the non-dimensional Sand_DBL_ number.** The data points indicate two regimes within the depicted range of Sand_DBL_ numbers. For 10 < Sand_DBL_ < 30 best fit was found for 0.043⋅Sand_DBL_^2.05^ (r-squared value 0.8, Supp. Eq. 1) and for 30 < Sand_DBL_ < 1000 best fit was found for 25.04⋅Sand_DBL_^0.20^ (r-squared value 0.5, Supp. Eq. 2). Below a Sand_DBL_ number of 10 no anoxic microenvironments are formed and for a Sand_DBL_ above 100 all microbial colonies are anoxic. In total 1764 model runs were included. We did not observe a difference of the power-laws for cases with consumption only and cases with consumption and production.


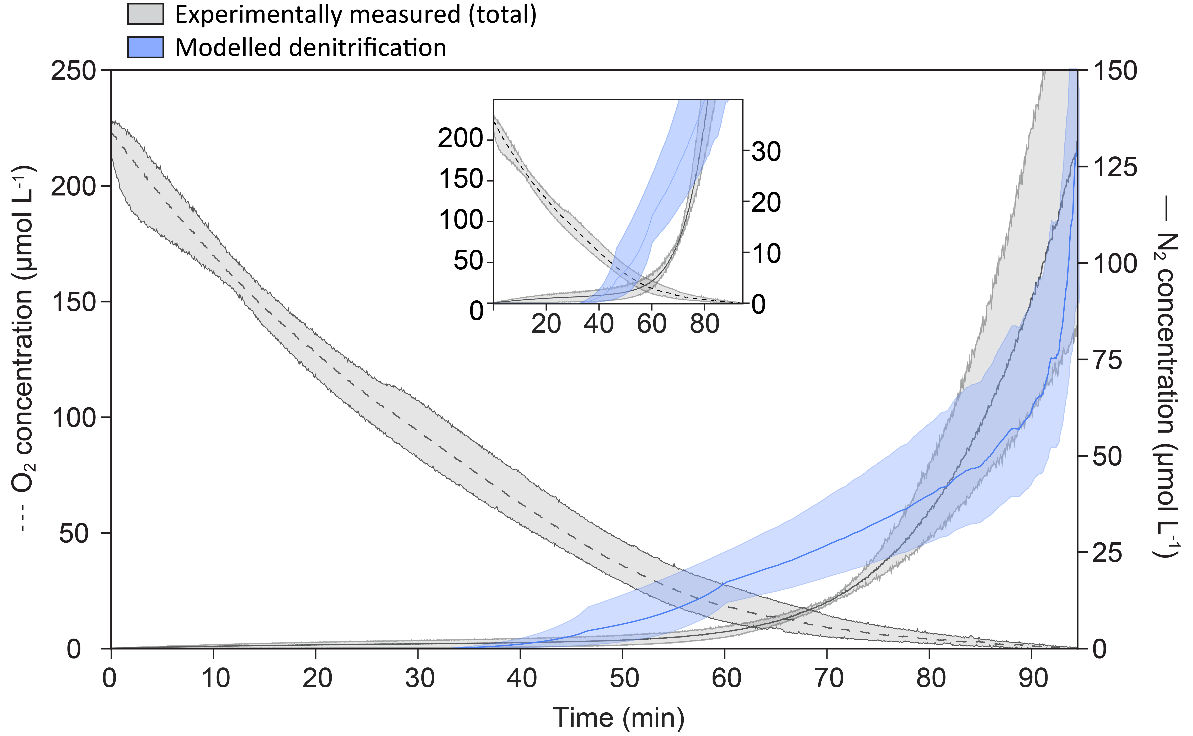


**Fig. S10.** **Denitrification attributed to anoxic microenvironments in sediment with bulk oxic porewater.** Co-occurring O_2_ respiration (dotted line) and N_2_ production (solid line) based on experimental measurements^22^ (grey) in sandy sediments. The data is compared to modelled N_2_ produced from the anoxic microenvironments (blue) based on the Sand_DBL_ relationship. The insert shows the same data with different y-axis limits to highlight the production of N_2_ at high O_2_ concentrations.


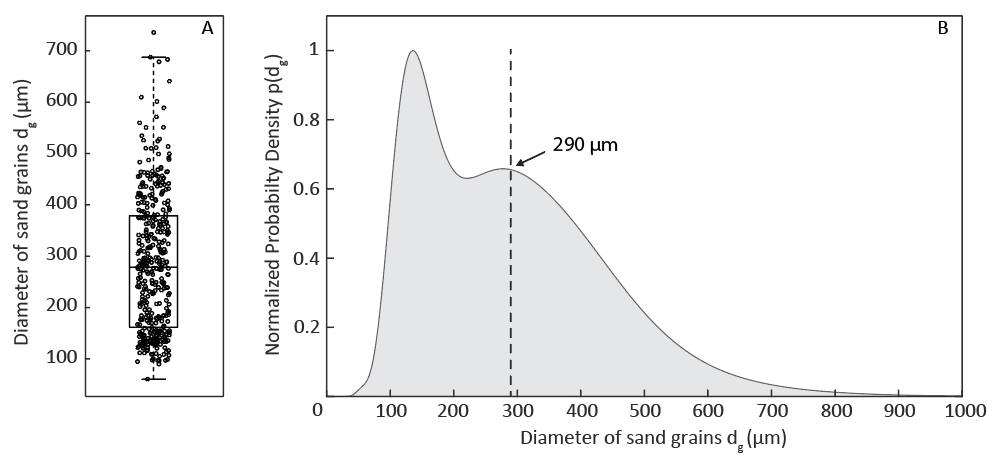


**Fig. S11.** **Sediment grain size distribution determined from photomicrographs**. **A** Variation in the size of the sand grains is represented using a box-whisker plot where the upper and lower sides are the quartiles, the line in the box represents the median, and the upper and lower line represent maximum and minimum excluding outliers. **B** Sand grain probability density distribution with the median at 290 µm (dashed line, n = 400).


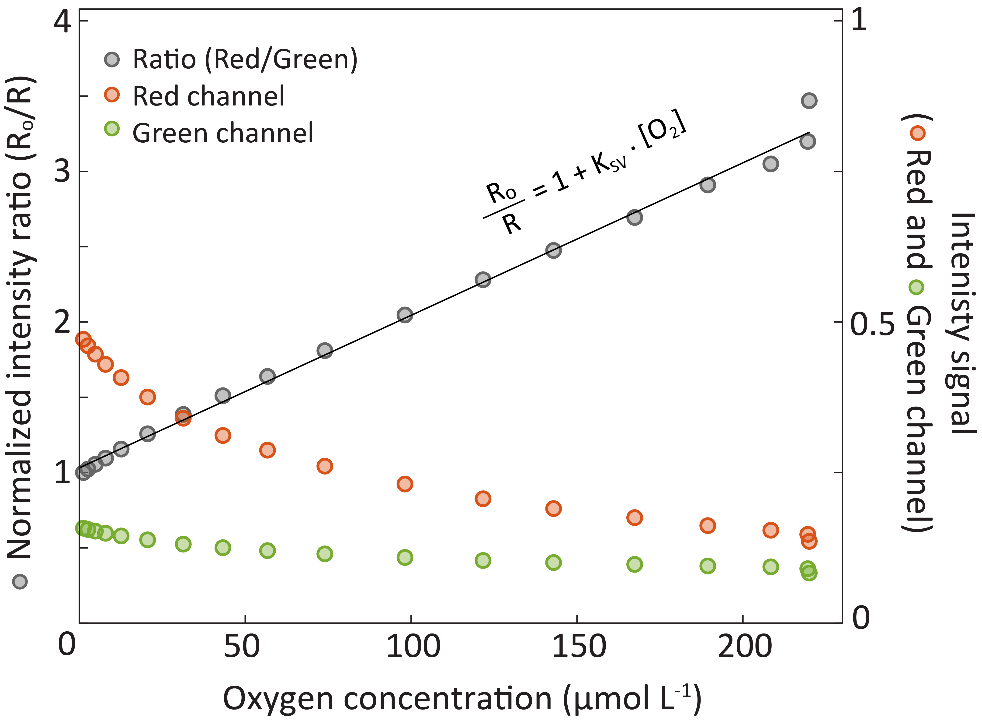


**Fig. S12.** **Calibration curve for sensor particles (K_SV_=0.01 and R_0_=2.99, R^2^=0.99).** Ratiometric imaging was performed using a RGB camera. The O_2_ -sensitive luminescence (red circles, red channel R) was referenced through an O_2_ -insensitive dye (green circles, green channel G, see also methods). The ratiometric signal (R/G) is shown in gray with solid line representing the best fit which was used for calibration.


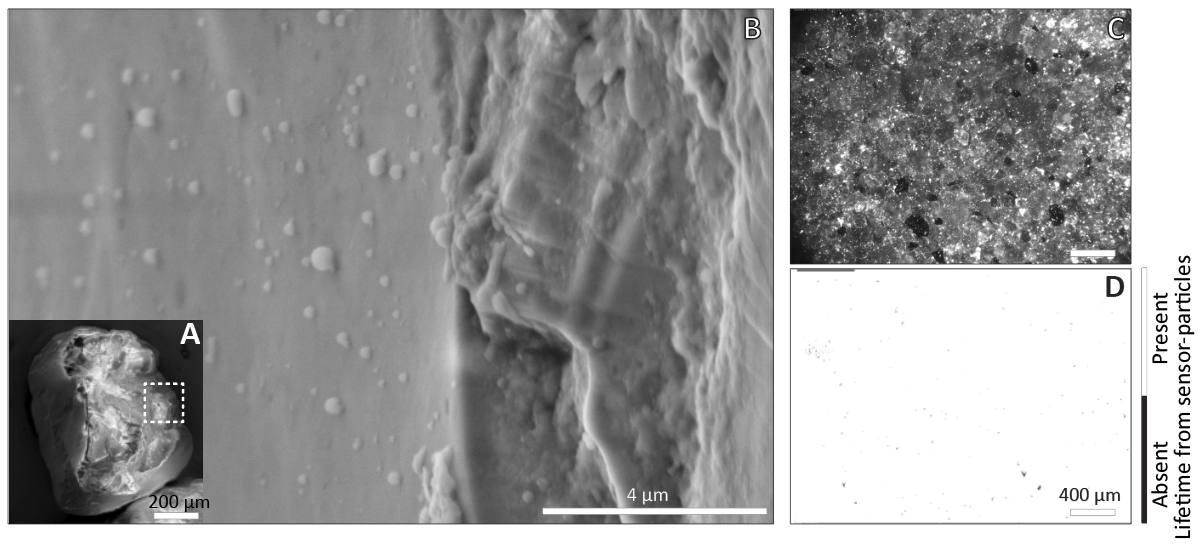


**Fig. S13.** **Scanning electron micrographs and life-time imaging of sensor particles coated on sand grains.** **A,B** Scanning electron micrograph of sand grains showing the distribution of sensor particles on the surface of a sand grain (~5 particles µm^-2^). We applied lifetime imaging to determine the presence of sensor particles on the sand grain surface, **C** intensity image (exc.: ~450 nm, em.: 500-550 nm) of sand grains with sensor particles, **D** binary image of the fluorescence lifetime where a threshold of 30 µs was selected. White pixels indicate a lifetime where sensor particles are present (lifetime > 30 µs) and black indicates areas where sensor particles were absent (< 30 µs).


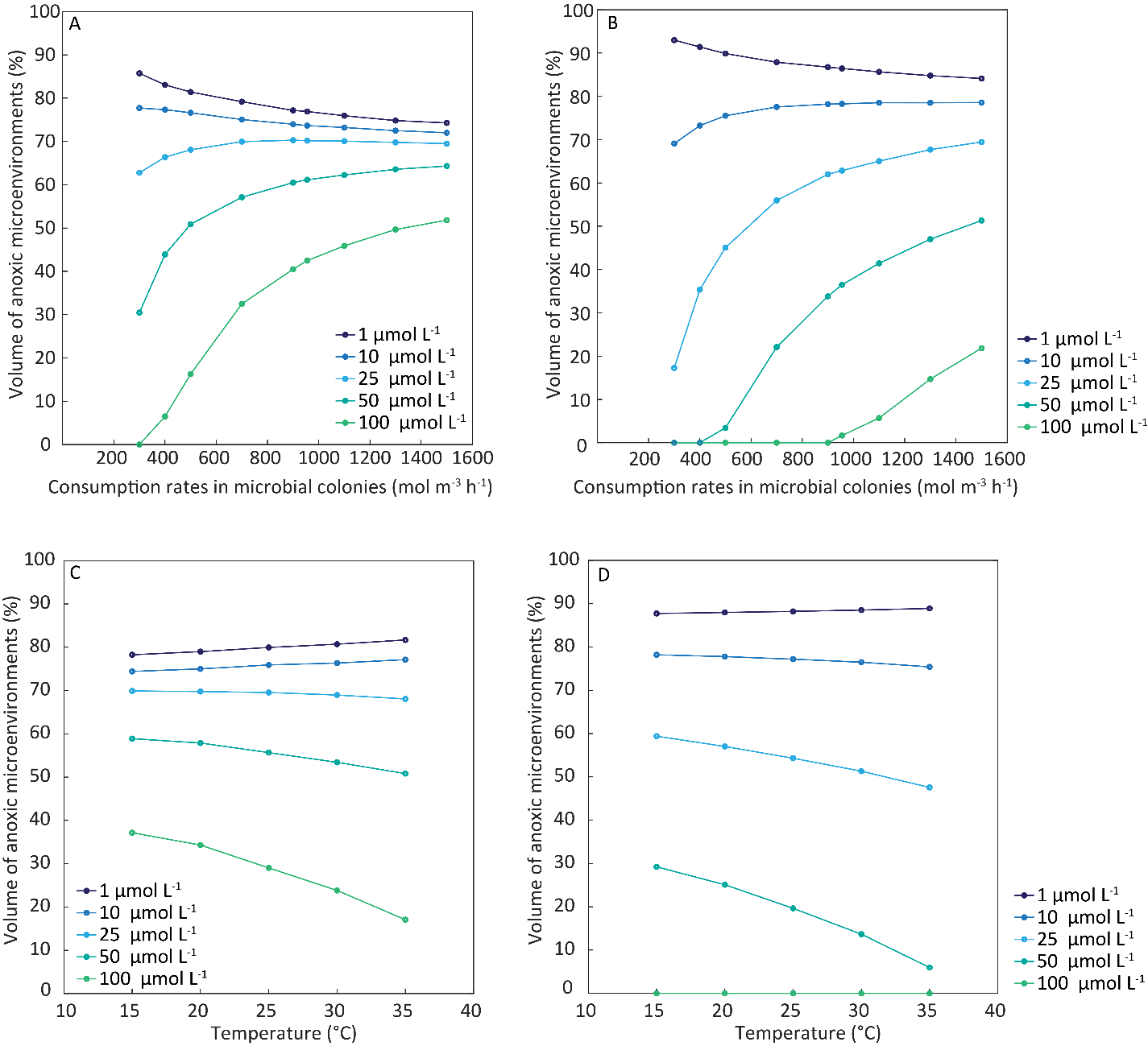


**Fig. S14.** **The effect of varying O_2_ consumption rates and temperature on the development of anoxic microenvironments.** **A** Variations of O_2_ consumption rates in the microbial colonies for a low pore water velocity (1 µm s^-1^) under five different inflow O_2_ concentrations. **B** Variations of O_2_ consumption rates at high pore water velocity (100 µm s^-1^) for five different inflow O_2_ concentrations. **C** Variation in the formation of anoxic microenvironments for different temperatures, taking into account the differences in the diffusion coefficient of O_2_, at low pore water velocity (1 µm s^-1^) for five different inflow O_2_ concentrations. **D** Variation in the formation of anoxic microenvironments for different temperatures, at higher pore water velocity (100 µm s^-1^) for five different inflow O_2_ concentrations.


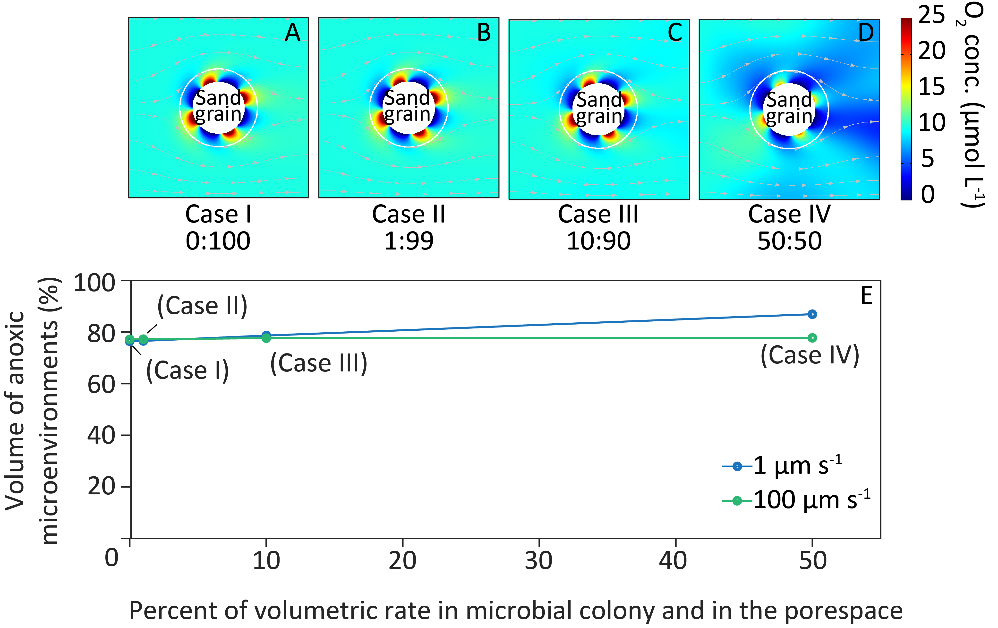


**Fig. S15.** **Variation in the volume of anoxic microenvironments due to different ratio of microbial respiration on the sand grain surface and in the porespace.** **A-D** Change in the concentration of O_2_ in the porespace due to changing percent of volumetric rate in the microbial colony and in the porespace within four different cases, namely, volumetric rate only on the sand surface (case I), 99% on the sand surface and 1% in porespace (case II), 10% on sand surface and 90% in porespace (case III), 50% on sand surface and 50% in porespace (case IV). **E** Change in the volume of anoxic microenvironments due to changing volumetric rates in the microbial colony and in the porespace.


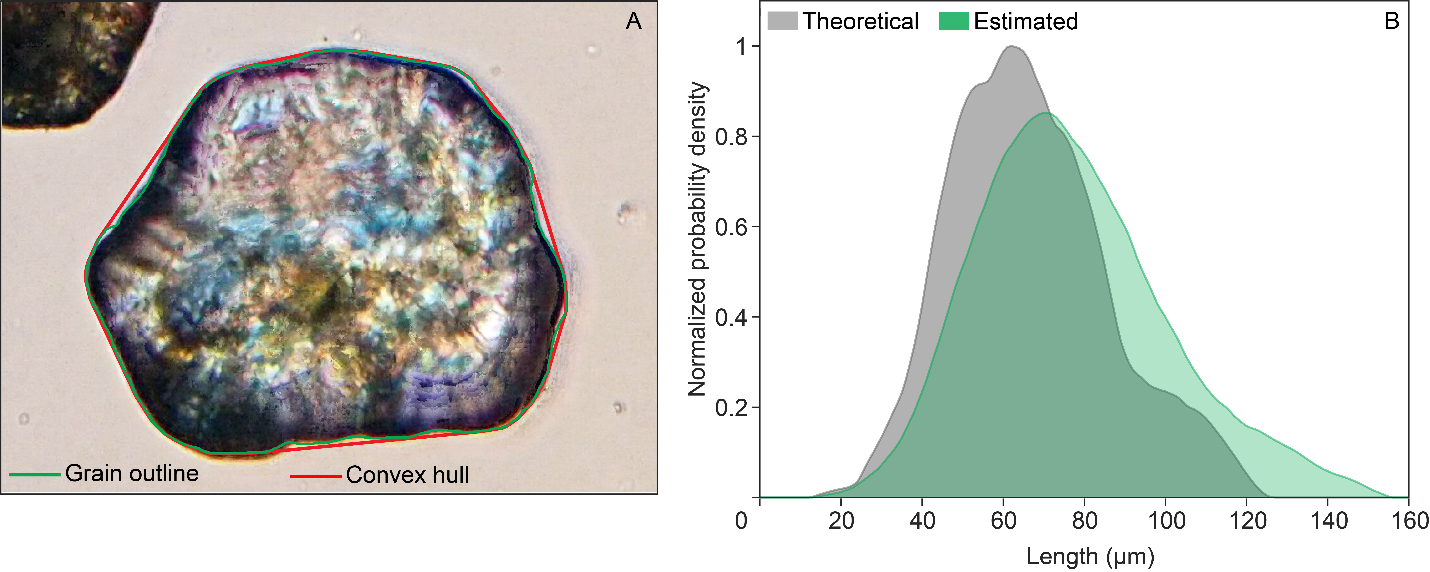


**Fig. S16**: **Effect of surface roughness on boundary layer formation and microenvironment development.** **A** Comparison of the actual sand grain outline (green) with the smoothed convex hull outline (red), illustrating surface roughness and irregularities. **B** Plot showing the theoretical diffusive boundary layer (gray) compared to the expanded boundary layer (green) induced by surface roughness, as estimated from the convex hull. The increased thickness highlights the potential for enhanced diffusion limitation and the development of microenvironments.

**Table S1.** Model parameters applied in eq. 4 to eq. 7.

| **Parameter** | **Equation/Values adopted** | **Description** |
| --- | --- | --- |
| D_O2_ | 2.0⋅10^-9^ m^2^ s^-1^ | Diffusion coefficient of O_2_ in water^23^ |
| D_N2_ | 2.6⋅10^-9^ m^2^ s^-1^ | Diffusion coefficient of N_2_ in water ^23^ |
| D_eps_ | 1.1⋅10^-9^ m^2^ s^-1^ | Diffusion coefficient of gas corrected for EPS/biofilm in the reactive zone ^23^ |
| d_g_ | 290 µm | Median diameter of sand grains |
| R_O2_ | 300 – 1500 mol m^-3^ h^-1^ | Volumetric O_2_ consumption rates |
| D | 1.8⋅10^-9^ to 3.0⋅10^-9^ m^2^ s^-1^ | Diffusion coefficients |
| vel | 0 - 500 µm s^-1^ | ^a^Pore water velocity^24,25^ |
| c_O2_ | 0 - 100 µmol L^-1^ | Inflow O_2_ concentration |
| K_m_ | 0.1 µmol L^-1^ | Half-saturation constant for O_2_ |
| cf | 0.1 | Ratio of aerobic respiration (AR) rates to denitrification (DN)^22^ |
| c_inh_ | 0.1 µmol L^-1^ | Inhibition constant for denitrification |
| ^a^Values adopted based on in situ measurements for bottom water velocity and pore water velocity | | |

**Table S2:** Compilation of literature values of measured denitrification rates on permeable sediments from globally distributed shelf ecosystems. The contribution of anoxic microenvironments in bulk oxic sandy sediments to total N-loss is calculated based on the Elliott model^26^ (see methods “Areal denitrification estimates” and eq. 10) and our Sand_DBL_ relationship (see methods “Relationships for the development of anoxic microenvironments”, Supp. Eq. 3). The numbers in brackets for “Contribution of anoxic microenvironments in oxic sandy sediments to total N-loss” denotes the error margin based on the uncertainty of the volumetric rates in the microbial colonies.

| **Location** | **Median Grain Sizes**  **(μm)** | **Bottom water**  **O_2_ (μmol L^-1^)** | **Bottom water Nitrate**  **(μmol L^-1^)** | **Bottom water velocities**  **(m s^-1^)** | **O_2_ Consumption Rates**  **(μmol L^-1^ h^-1^)** | **Denitrification Rates**  **(μmol L^-1^ N h^-1^)** | **Contribution of anoxic microenvironments in oxic sandy sediments to total N-loss** |  |
| --- | --- | --- | --- | --- | --- | --- | --- | --- |
| South Atlantic Bight^19^ | 200-700 | NA ^f^ | NA ^f^ | 0.1-0.2 ^a^ | 3.8-16.6 | 0.04 – 0.9 | 8-62 % (15%) |  |
| North Sea^18^ | 342-576 | NA ^f^ | 6-7 | 0.2 | 15- 250 | 0.9 – 6.8 | 26-56 % (5 %) |  |
| Gulf of Mexico^27^ | 170-250^b^ | NA ^f^ | 0.4-4.8 | NA ^f^ | 2.7-11.8 ^e^ | 0.3 – 1.2 ^c^ | 31-35 % (20 %) |  |
| Beach face, Sanggou Bay, China^28^ | 199-204 | 30.6-101 | 10.2-80.4 | NA ^f^ | 5 - 25 | 2.3 - 4.3 | 14-30 % (22 %) |  |
| Victoria, Australia^16^ | 550 ^d^ | NA ^f^ | 1.9-4.9 | NA ^f^ | 36-207 | 0.9 - 7.5 | 14-47 % (6 %) |  |
| North West African Shelf^24^ | 102-513 | 55.9-81.0 | 19.4-30.3 | 0.2 | 12-118 ^e^ | 1.2 – 11.8 | 22-50 % (4 %) |  |

^a^ Adopted bottom water velocities^24^

^b^ Grain size was calculated based on the permeabilities^29^

^c^ Volumetric denitrification rates were estimated by dividing chamber incubation fluxes by nitrate penetration depths

^d^ Grain size was estimated from a study^30^ that took place in the same region

^e^ O_2_ consumption rates were calculated by using a constant ratio between volumetric denitrification and O_2_ consumption of 1:10

^f^ If not available, we assumed bottom water velocities of 0.1 m s^-1^, nitrate concentrations of 5 μmol L^-1^, O_2_ air saturation was calculated based on the temperature and salinity at the study site

**Supporting Information References**

1. Mendoza-Lera, C. *et al.* Importance of advective mass transfer and sediment surface area for streambed microbial communities. *Freshw Biol* **62**, 133–145 (2017).

2. Musat, N. *et al.* Microbial community structure of sandy intertidal sediments in the North Sea, Sylt-Rømø Basin, Wadden Sea. *Syst Appl Microbiol* **29**, 333–348 (2006).

3. Rusch, A., Forster, S. & Huettel, M. *Bacteria, Diatoms and Detritus in an Intertidal Sandflat Subject to Advective Transport across the Water-Sediment Interface*. *Biogeochemistry* vol. 55 (2001).

4. Neumann, A., Möbius, J., Hass, H. C., Puls, W. & Friedrich, J. Empirical model to estimate permeability of surface sediments in the German Bight (North Sea). *J Sea Res* **127**, 36–45 (2017).

5. Jørgensen, B. B. Bacterial sulfate reduction within reduced microniches of oxidized marine sediments. *Mar Biol* **41**, 7–17 (1977).

6. Coyte, K. Z., Tabuteau, H., Gaffney, E. A., Foster, K. R. & Durham, W. M. Microbial competition in porous environments can select against rapid biofilm growth. *Proceedings of the National Academy of Sciences* **114**, E161–E170 (2017).

7. Aufrecht, J. A. *et al.* Pore-scale hydrodynamics influence the spatial evolution of bacterial biofilms in a microfluidic porous network. *PLoS One* **14**, e0218316 (2019).

8. Kurz, D. L. *et al.* Competition between growth and shear stress drives intermittency in preferential flow paths in porous medium biofilms. (2022) doi:10.1073/pnas.

9. Ceriotti, G., Borisov, S. M., Berg, J. S. & De Anna, P. Morphology and Size of Bacterial Colonies Control Anoxic Microenvironment Formation in Porous Media. *Environ Sci Technol* **56**, 17471–17480 (2022).

10. Lehto, N., Glud, R. N., á Nordi, G., Zhang, H. & Davison, W. Anoxic microniches in marine sediments induced by aggregate settlement: Biogeochemical dynamics and implications. *Biogeochemistry* **119**, 307–327 (2014).

11. Drescher, K., Shen, Y., Bassler, B. L. & Stone, H. A. Biofilm streamers cause catastrophic disruption of flow with consequences for environmental and medical systems. *Proc Natl Acad Sci U S A* **110**, 4345–4350 (2013).

12. Scheidweiler, D., Peter, H., Pramateftaki, P., de Anna, P. & Battin, T. J. Unraveling the biophysical underpinnings to the success of multispecies biofilms in porous environments. *ISME Journal* **13**, 1700–1710 (2019).

13. Franklin, S., Vasilas, B. & Jin, Y. More than Meets the Dye: Evaluating Preferential Flow Paths as Microbial Hotspots. *Vadose Zone Journal* **18**, 1–8 (2019).

14. Matyka, M., Khalili, A. & Koza, Z. Tortuosity-porosity relation in porous media flow. *Phys Rev E Stat Nonlin Soft Matter Phys* **78**, (2008).

15. Ahmerkamp, S. *et al.* The effect of sediment grain properties and porewater flow on microbial abundance and respiration in permeable sediments. *Sci Rep* **10**, (2020).

16. Evrard, V., Glud, R. N. & Cook, P. L. M. The kinetics of denitrification in permeable sediments. *Biogeochemistry* **113**, 563–572 (2013).

17. Marchant, H. K., Lavik, G., Holtappels, M. & Kuypers, M. M. M. The fate of nitrate in intertidal permeable sediments. *PLoS One* **9**, (2014).

18. Marchant, H. K. *et al.* Coupled nitrification-denitrification leads to extensive N loss in subtidal permeable sediments. *Limnol Oceanogr* **61**, 1033–1048 (2016).

19. Rao, A. M. F., McCarthy, M. J., Gardner, W. S. & Jahnke, R. A. Respiration and denitrification in permeable continental shelf deposits on the South Atlantic Bight: Rates of carbon and nitrogen cycling from sediment column experiments. *Cont Shelf Res* **27**, 1801–1819 (2007).

20. Santos, I. R., Eyre, B. D. & Glud, R. N. Influence of porewater advection on denitrification in carbonate sands: Evidence from repacked sediment column experiments. *Geochim Cosmochim Acta* **96**, 247–258 (2012).

21. Rao, A. M. F., McCarthy, M. J., Gardner, W. S. & Jahnke, R. A. Respiration and denitrification in permeable continental shelf deposits on the South Atlantic Bight: N2:Ar and isotope pairing measurements in sediment column experiments. *Cont Shelf Res* **28**, 602–613 (2008).

22. Marchant, H. K. *et al.* Denitrifying community in coastal sediments performs aerobic and anaerobic respiration simultaneously. *ISME Journal* **11**, 1799–1812 (2017).

23. Stewart, P. S. Diffusion in Biofilms. *J Bacteriol* **185**, 1485–1491 (2003).

24. Reimers, C. E. *et al.* In situ measurements of advective solute transport in permeable shelf sands. *Cont Shelf Res* **24**, 183–201 (2004).

25. Ahmerkamp, S. *et al.* Regulation of benthic oxygen fluxes in permeable sediments of the coastal ocean. *Limnol Oceanogr* **62**, 1935–1954 (2017).

26. Elliott, A. H. & Brooks, N. H. Transfer of nonsorbing solutes to a streambed with bed forms: Theory. *Water Resour Res* **33**, 123–136 (1997).

27. Gihring, T. M., Lavik, G., Kuypers, M. M. M. & Kostka, J. E. Direct determination of nitrogen cycling rates and pathways in Arctic fjord sediments (Svalbard, Norway). *Limnol Oceanogr* **55**, 740–752 (2010).

28. Jiang, S. *et al.* Response of Nitrate Processing to Bio-labile Dissolved Organic Matter Supply Under Variable Oxygen Conditions in a Sandy Beach Seepage Face. *Front Mar Sci* **8**, (2021).

29. Gangi, A. F. Variation of whole and fractured porous rock permeability with confining pressure. *International Journal of Rock Mechanics and Mining Sciences & Geomechanics Abstracts* **15**, 249–257 (1978).

30. Chen, Y. J. *et al.* Metabolic flexibility allows bacterial habitat generalists to become dominant in a frequently disturbed ecosystem. *ISME Journal* **15**, 2986–3004 (2021).
